# *pelo* Is Required for High Efficiency Viral Replication

**DOI:** 10.1371/journal.ppat.1004034

**Published:** 2014-04-10

**Authors:** Xiurong Wu, Wan-Ting He, Shuye Tian, Dan Meng, Yuanyue Li, Wanze Chen, Lisheng Li, Lili Tian, Chuan-Qi Zhong, Felicia Han, Jianming Chen, Jiahuai Han

**Affiliations:** 1 State Key Laboratory of Cellular Stress Biology, Innovation Center for Cell Biology, School of Life Sciences, Xiamen University, Xiamen, Fujian, China; 2 The Key Laboratory of Marine Biogenetic Resources, The Third Institute of Oceanography, State Oceanic Administration of China, Xiamen, Fujian, China; Stanford University, United States of America

## Abstract

Viruses hijack host factors for their high speed protein synthesis, but information about these factors is largely unknown. In searching for genes that are involved in viral replication, we carried out a forward genetic screen for Drosophila mutants that are more resistant or sensitive to *Drosophila C virus* (DCV) infection-caused death, and found a virus-resistant line in which the expression of *pelo* gene was deficient. Our mechanistic studies excluded the viral resistance of *pelo* deficient flies resulting from the known *Drosophila* anti-viral pathways, and revealed that *pelo* deficiency limits the high level synthesis of the DCV capsid proteins but has no or very little effect on the expression of some other viral proteins, bulk cellular proteins, and transfected exogenous genes. The restriction of replication of other types of viruses in *pelo* deficient flies was also observed, suggesting *pelo* is required for high level production of capsids of all kinds of viruses. We show that both *pelo* deficiency and high level DCV protein synthesis increase aberrant 80S ribosomes, and propose that the preferential requirement of *pelo* for high level synthesis of viral capsids is at least partly due to the role of *pelo* in dissociation of stalled 80S ribosomes and clearance of aberrant viral RNA and proteins. Our data demonstrated that *pelo* is a host factor that is required for high efficiency translation of viral capsids and targeting *pelo* could be a strategy for general inhibition of viral infection.

## Author Summary

Viruses often can highly efficiently utilize the host system to make huge amounts of viral proteins for their replication; however, which host factors are needed by the viruses are largely unknown. We analyzed about one hundred *Drosophila* mutants and found that the *pelo* mutation in *Drosophila* inhibited *Drosophila C virus* (DCV) replication. We found *pelo* is specifically required for high efficiency synthesis of proteins of a number of viruses, suggesting that inhibition of *pelo* may mediate a general antiviral activity. We proposed that the function of *pelo* in quality control of protein synthesis is required for high efficiency viral protein synthesis. Our study presented here identifies a new host factor, *pelo*, that is specifically required for effective viral replication and it may be a new potential therapeutic target for broad-spectrum antiviral therapy.

## Introduction

Viruses are the most abundant intracellular pathogens on the earth. They can infect all living organisms and hijack their host factors for replication [1]. In order to withstand virus infections, their hosts have evolved multiple antiviral defense mechanisms [2], [3], [4]. For many years, scientists have been studying host-virus interactions in order to develop new and more effective strategies for the prevention and treatment of viral infection.

*Drosophila melanogaster* has been shown to be a powerful model system in studying host-pathogen interactions [5]. In addition to the widely appreciated achievement in studying antibacterial and antifungal immunity by using *Drosophila* [6], there is also growing understanding of *Drosophila*-virus interactions [7], [8]. Several antiviral innate immunity pathways and their corresponding molecular mechanisms have been deciphered in *Drosophila*. RNA interference is the major antiviral pathway in *Drosophila* [9]. Similar to plants, *Drosophila* can generate both a local and a systemic antiviral RNAi response [10]. However, viruses can counteract host RNAi defense by expressing viral suppressors of RNAi (VSRs), such as FHV-B2, DCV-1A, and Crpv-A [11], [12], [13], [14]. Inducible gene expressions in response to viral infection also contribute to antiviral immunity, including the JAK-STAT pathway and the DExD/H-box helicase Dicer-2-mediated antiviral gene induction [15], [16]. The Toll and IMD pathways are involved in restricting some specific viruses by mechanisms yet to be clarified [17], [18]. Autophagy plays an important antiviral role against the vesicular stomatitis virus (VSV) in *Drosophila*, which is initiated by Toll-7 after its recognition of the VSV glycoprotein VSV-G [19], [20]. It is apparent that some antiviral mechanisms are conserved between *Drosophila* and mammals but others are uniquely present in *Drosophila*.

Hijacking host cellular machineries for viral replication is another major part of host-virus interaction [1], [21]. Studies in *Drosophila* have revealed that the clathrin-mediated endocytotic pathway is required for viral entry [22], some ribosomal proteins are involved in viral IRES-dependent translation [23], and the coat protein complex I (COPI) coatamer and fatty acid biosynthesis are required to form the intracellular vesicular compartment for viral replication [24]. All of these appear to be commonly involved in host-virus interaction of many different viruses. Targeting these events might be able to interfere with the replication of a broad panel of viruses; however, achieving the goal of virus inhibition without affecting normal cell function is a challenging task. Without exception, viruses have to use cellular protein translation machinery to synthesize their proteins. In many cases, viruses take over the hosts' protein synthesis machinery to make huge amounts of viral proteins for their replication [25]. How can viruses so highly efficiently utilize the cellular system to synthesize their proteins is largely unknown. Information on the cellular factors that are required for high speed synthesis of viral proteins is very limited.

Protein translation is a tightly controlled cellular process, and also is a part of the checking mechanism that eliminates aberrant transcripts and proteins [26]. The pelo-Hbs1 complex (also known as Dom34-Hbs1) recognizes stalled ribosomes caused by defective mRNAs as well as rRNAs and promotes ribosomal subunit dissociation and the release of peptidyl-tRNA [27], [28]. Therefore, the complex participates in quality control for non-go decay (NGD) and non-stop decay (NSD) [29], [30], [31]. ABCE1, a conserved member of the ATP-binding cassette (ABC) family of proteins, is involved in pelo-Hbs1 mediated disassembly of the ribosome in mammalian cells [32]. Recent work has shown that Dom34-Hbs1 is also required for nonstop protein clearance from translocators for normal organelle protein influx [33]. *pelo* is a highly conserved gene from yeast to human. It contains three eRF1 (eukaryotic translation termination factor 1) domains. X-ray structural analysis of yeast Dom34 and the archaea homolog Pelota reveals that the structure of pelo is similar to eRF1 except for its N-terminal domain [34], [35]. Deletion of Dom34 in yeast led to delayed progression through the G1 phase of the cell cycle, aberrant meiosis, and an altered polyribosome profile [36], [37]. In *Drosophila*, *pelo* is required for meiotic cell division and controls germ-line stem cell self-renewal [38], [39]. The mammalian homolog of *pelo* may be required for progression of the mitotic cell cycle and *pelo* deficient mice are embryonic lethal [40].

*Drosophila C virus* (DCV) is the best studied and a relatively simple *Drosophila* virus. It belongs to the *Dicistroviridae* family. It is a non-enveloped RNA virus and its capsid is composed of the three major proteins VP1 (33 kDa), VP2 (29 kDa), VP3 (28 kDa), and two minor proteins, VP0 (37.7 kDa) and VP4 (8.5 kDa), with VP0 as a precursor of VP3 and VP4 [41]. DCV contains only one single positive-strand RNA genome which is polyadenylated and with a genome-linked protein at the 5′end [42], [43]. DCV replicates rapidly after injection into adult flies and causes host death in as few as 3 days making it an ideal pathogen for performing a death screen [44]. In an effort to search for host factors that are required for the viral replication, we carried out a forward genetic screen for *Drosophila* mutants that are more resistant or sensitive to DCV infection-caused death. As a result, we found that *pelo* deficiency can mediate DCV resistance. Further characterization revealed that *pelo* is not involved in the known antiviral pathways in *Drosophila*. Our mechanistic studies showed that *pelo* is required for high efficiency synthesis of DCV capsid proteins and thus DCV replication; the function of *pelo* in the dissociation of stalled 80S ribosomes is at least part of the underlying mechanism that allows for high speed synthesis of DCV capsid proteins.

## Results

### *pelo* deficiency in *Drosophila* mediates resistance to DCV infection-caused death

**Figure 1:**
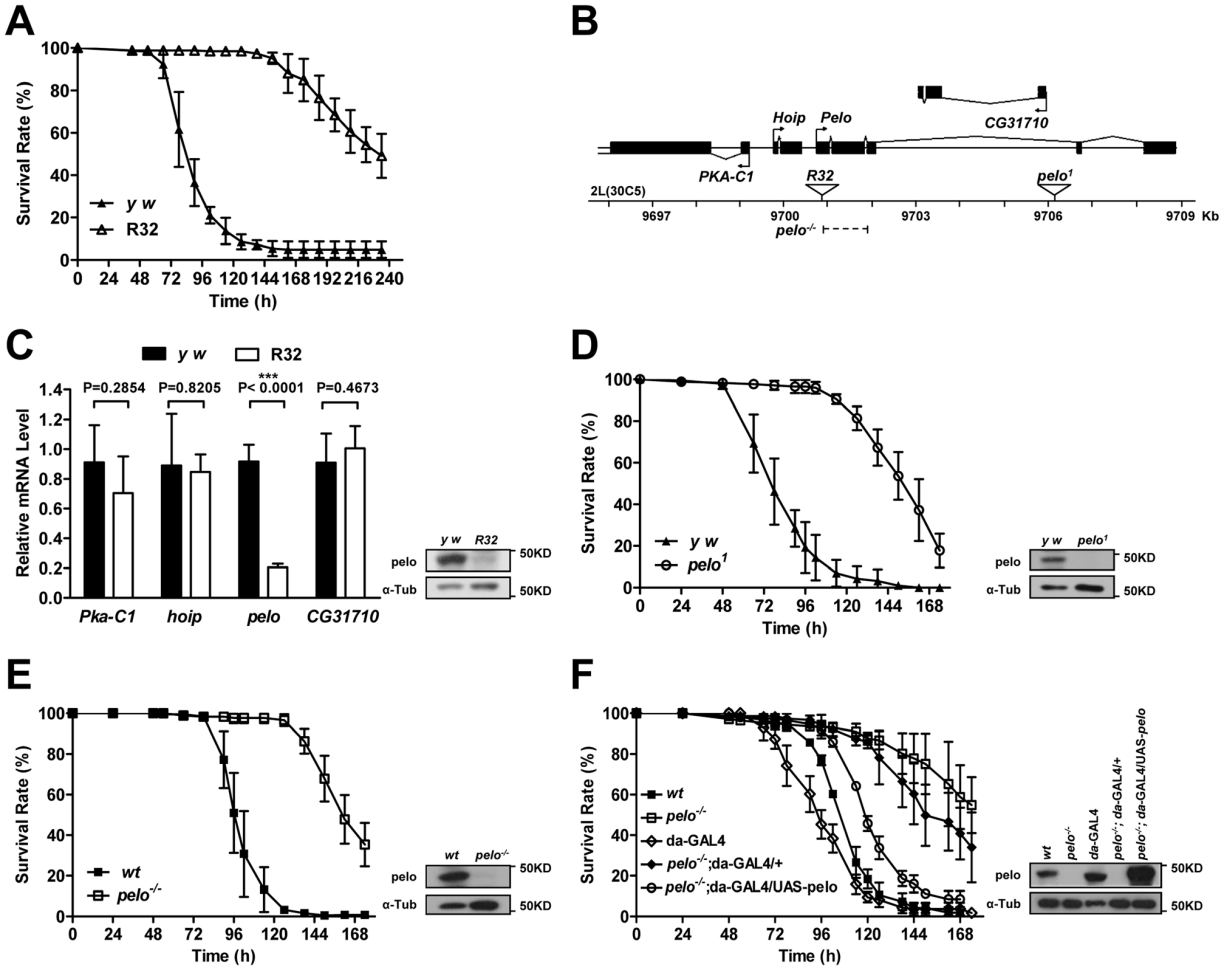
*pelo* deficiency mediates DCV resistance in adult flies. (**A**) *R32* is resistant to DCV infection. 2–4 days old flies were injected in the thorax with DCV and then monitored every 8 hours for mortality. *y w* was used as a genetic background control. Results are the mean ± SD of three independent experiments with 60 flies in each group. (**B**) Schematic diagram of the pelo deficient flies. The triangles represent the P-element insertion site in R32 and pelo^1^ flies respectively. The dashed line shows the deletion region in pelo^−/−^. (**C**) Relative mRNA levels of *Pka-C1*, *hoip*, *pelo*, and *CG31710* were measured by qRT-PCR. Expression levels of each gene were normalized to *Rp49* and shown as the relative values compared to y w. Data are the mean ± SD of three independent experiments. The protein expression levels of pelo were measured by immunoblotting with anti-pelo antibody and α-Tub (α-tubulin) was used as a loading control. (**D–F**) Survival curve of flies of indicated genotype after DCV infection. Experiment was performed as described in **A**. pelo^1^ was backcrossed into the y w genetic background. pelo^−/−^ was generated by imprecise excision of the P-element from the line R32 and a precise P-element excision line was used as the wild-type (wt) control.

To identify genes that are involved in host-virus interactions, we screened about 100 P-element insertion fly lines, generated by mobilizing a P {Mae-UAS.6.11} transposon to random autosomal sites from the X chromosome, for their sensitivity to DCV-induced death. The mutants were infected by septic injury with DCV to identify lines that are more resistant or sensitive to DCV infection than wild-type. The survival curve of a group of mutant lines is shown as an example of the screen (Figure S1). Two virus-resistant lines were screened out and we were able to identify the P-element insertion site in one of the lines numbered R32. The resistance to DCV induced death of R32 is shown in Figure 1A. The P-element insertion site of *R32* is in the 5′UTR of the *pelo* gene and thus may disrupt *pelo* expression (Figure 1B). Since the genes of *Pka-C1*, *hoip*, and *CG31710* are also located near the P-element insertion site, we used qRT-PCR to determine the mRNA expression levels of these and the *pelo* genes. We found that the expression of *pelo* is reduced while the other three genes are not changed in the *R32* line (Figure 1C). We then generated a specific polyclonal antibody to pelo and used it to confirm that the protein level of pelo was really reduced in *R32* (Figure 1C).

*R32* fly is healthy but male-sterile, which is consistent with a previously reported *pelo* deficient line called *pelo^1^* [38], [39]. *pelo^1^* has a P-element inserted in the third intron of *pelo* (Figure 1B), which disrupts pelo protein expression (Figure 1D). To collect more evidence for or against the role of *pelo* in DCV sensitivity, we compared the sensitivity of *pelo^1^* and its control line to DCV infection. *pelo^1^* is resistant to DCV infection (Figure 1D), supporting the conclusion obtained from the *R32* line that *pelo* deficiency causes DCV resistance. In order to better study the role of *pelo* in DCV sensitivity, we generated a *pelo* knockout line by deleting exons 1 and 2 (Figure 1B), which completely eliminated pelo protein expression (Figure 1E). Consistently, the *pelo^−/−^* line had DCV resistant phenotype (Figure 1E). To unambiguously demonstrate that *pelo* deficiency causes DCV resistance in *Drosophila*, we rescued the expression of *pelo* in *pelo^−/−^* flies by using a UAS-*pelo* transgene and a ubiquitous *da-GAL4* driver (Figure 1F). The rescued line became more sensitive to DCV infection and thus had a phenotype similar to wild-type flies (Figure 1F). It is reported that bacterial symbiont *Wolbachia* increases resistance of *Drosophila* to RNA viral infections [45]. To examine whether *Wolbachia* infection influences our experiments, we measured *Wolbachia* infection status in our fly lines. All the fly lines we used except *pelo^1^* are infected with *Wolbachia* (Figure S2A) and the infection levels are almost the same (Figure S2B), suggesting that the DCV resistant phenotype in *pelo* deficient flies is not caused by *Wolbachia* infection. Altogether, these data demonstrated that *pelo* deficiency results in DCV resistance in *Drosophila*.

### *pelo* deficiency inhibits DCV replication in adult flies and *Drosophila* S2 cells

**Figure 2:**
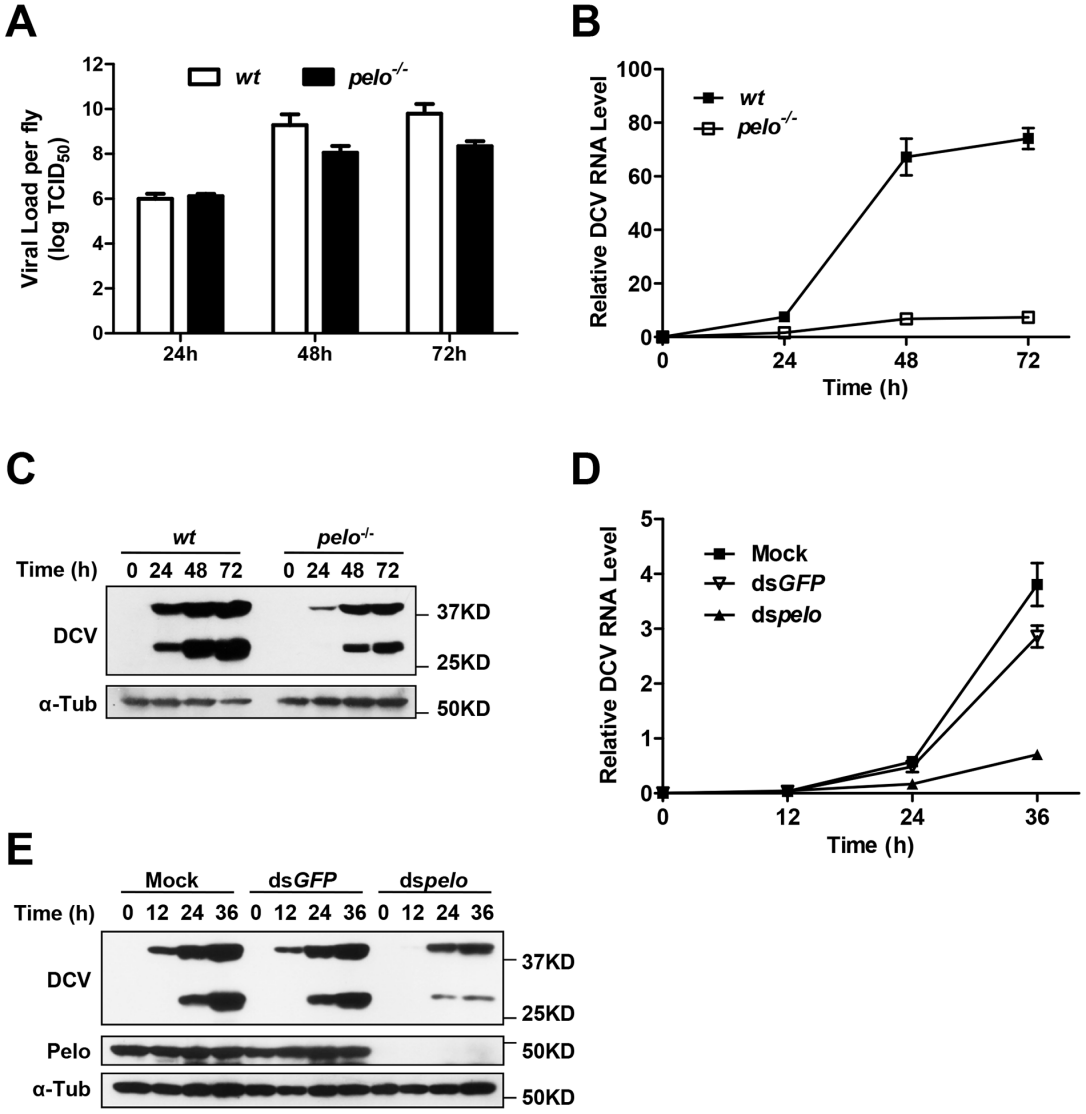
*pelo* deficiency inhibits viral replication. (**A–C**) Flies were challenged with DCV and then three pools of ten flies were collected at the indicated time points post-infection. The viral titer was determined by end-point dilution (**A**). The accumulation of DCV RNA was measured by qRT-PCR. Data represent the mean ± SD of triplicates (**B**). The accumulation of DCV capsid proteins was measured by immunoblotting with anti-DCV antibody (**C**). (**D** and **E**) S2 cells were untreated (Mock) or treated with dsRNAs against *GFP* (ds*GFP*) or *pelo* (ds*pelo*) for 6 days and then infected with DCV (MOI = 0.1). The accumulation of DCV RNA (**D**) or capsid proteins (**E**) was measured as described in **B** and **C**, respectively.

To characterize the *pelo* deficiency-mediated antiviral effect, we measured the viral titer at different time points in the virus-infected wild-type and *pelo^−/−^* flies before their death and found that the titer in the mutant flies were dramatically reduced compared to wild-type flies (Figure 2A). Consistent with the lower viral titer in *pelo^−/−^* flies (Figure 2A), the amounts of viral RNA and capsid proteins were also reduced in *pelo* ^−/−^ flies (Figures 2B and 2C). The antibody of DCV was produced by inoculation of rabbit with the purified virus, so that it can recognize all the viral capsid proteins. Based on molecular weight and mass spectrum analysis described later, we knew that the upper band is VP0 and the lower band is the mixture of VP1, VP2, and VP3. We also could detect a weak band of a molecular mass of 20 KD when in long exposure but not the 8.5 KD band corresponding to VP4 (data not shown). This decreased viral load correlated with the increased survival rate of the *pelo* mutant at later stages of DCV infection (Figure 1E). Thus, *pelo* deficiency-caused resistance to DCV infection-induced death is due to the fact that *pelo* deficiency limits DCV replication.

In order to study *pelo* deficiency-mediated resistance to DCV infection at the cellular level, we evaluated whether *Drosophila* S2 cells can be used in this study. *pelo* expression can be effectively knocked-down by RNAi in S2 cells (Figure 2E). *pelo* knockdown S2 cells have apparently normal morphology and do not have any noticeable changes in cell proliferation when compared with mock treated or control RNAi (ds*GFP*) treated S2 cells (Figure S3). We infected the control and *pelo* knockdown S2 cells with DCV and measured the viral RNA by qRT-PCR and the viral proteins by antibodies against viral capsid proteins. As shown in Figures 2D and 2E, both viral RNA and capsid proteins in *pelo* knockdown cells were much less than that in control cells. Thus the S2 cell line is a suitable culture cell system for studying the mechanism of *pelo* deficiency-mediated inhibition of DCV replication.

### *pelo* is not involved in the known *Drosophila* antiviral mechanisms

**Figure 3:**
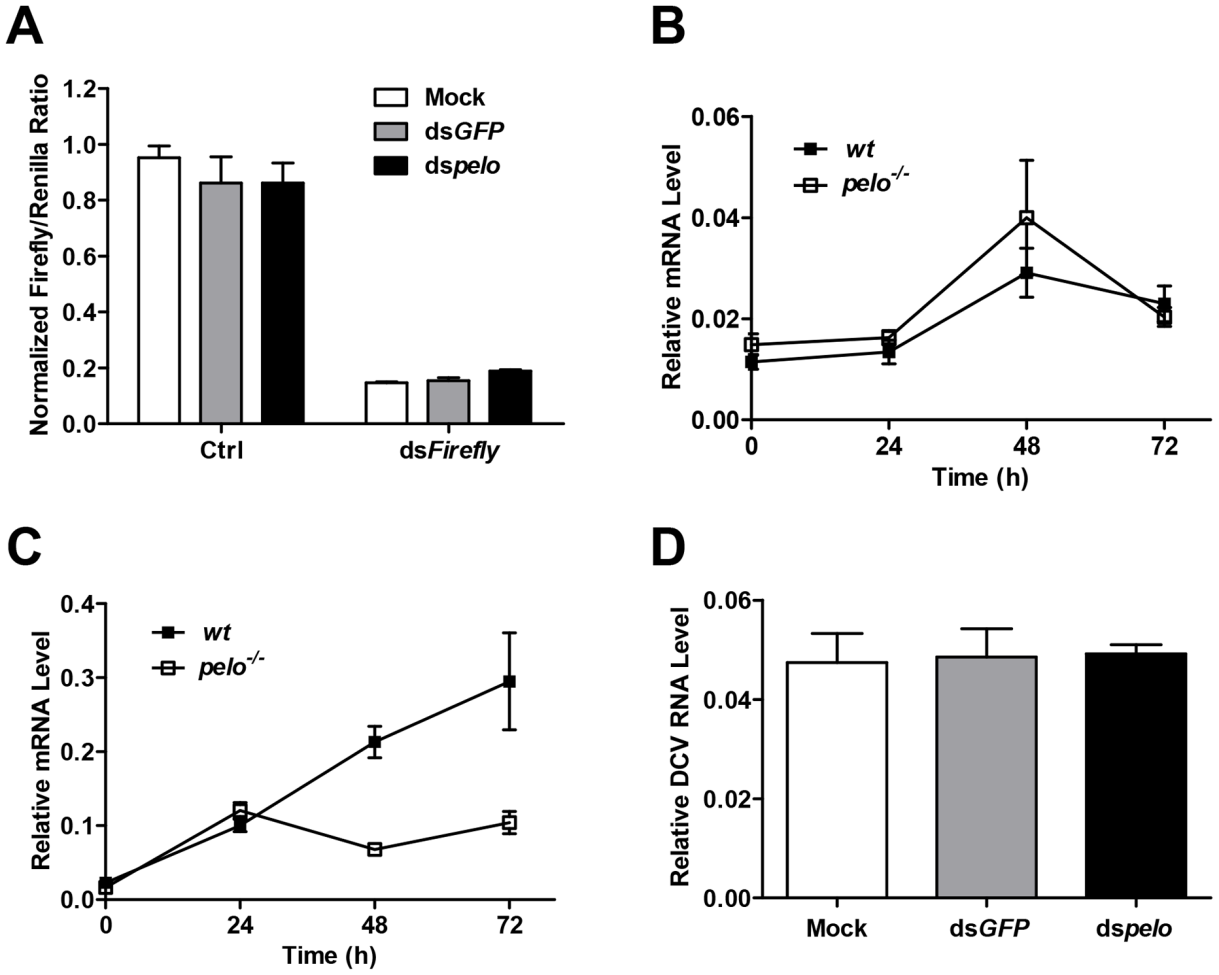
*pelo* deficiency has no effect on siRNA-mediated gene silencing, Dicer-2 or JAK-STAT mediated antiviral gene expression, and the entry of DCV into S2 cells. (**A**) Cells were treated with the indicated dsRNAs for 6 days and then co-transfected with reporter plasmids encoding Firefly and Renilla luciferase. 8 hours after transfection, cells were treated with dsRNA targeting the Firefly luciferase (dsFireflly) or were untreated as a control (Ctrl). Luciferase activities were assayed at 24 hours after dsRNA treatment. Ratios of Firefly to Renilla luciferase are shown. (**B** and **C**) Flies were infected with DCV and collected at the indicated time points post-infection. The inductions of the Dicer-2 mediated gene Vago (**B**) and JAK-STAT target gene Vir-1 (**C**) were analyzed by qRT-PCR. (**D**) Cells pretreated with the indicated dsRNAs were infected with DCV (MOI = 100) for 2 hours in the presence of cycloheximide (10 mg/ml) to prevent new synthesis of proteins. Viral uptake was measured by qRT-PCR analysis of internalized DCV genomic RNA. Data are the mean ± SD of triplicates.

There are several antiviral mechanisms in *Drosophila*, including RNAi, autophagy, antiviral gene expression regulated by Dicer-2, JAK-STAT, and NF-KB [8]. Since reported studies showed that the NF-KB pathway and autophagy can restrict certain viruses but not DCV in *Drosophila* [17], [18], [19], we explored whether *pelo* can work as a negative regulator of the other antiviral mechanisms. Because RNAi is the major defense mechanism against viral infection in *Drosophila*, we first tested whether loss of *pelo* affects siRNA-mediated gene silencing. S2 cells were co-transfected with *Firefly* and *Renilla* luciferase reporters and then treated with dsRNA targeting the *Firefly* luciferase. RNAi efficiency was measured by the decrease in *Firefly* luciferase activity relative to that of the control *Renilla* luciferase. We found that *pelo* deficiency had no effect on dsRNA-mediated inhibition of *Firefly* luciferase expression (Figure 3A). It is known that the induction of *Vago* by DCV infection is dependent on the Dicer-2 pathway [16] and we found that *pelo* deficiency did not further up-regulate *Vago* in DCV infected flies (Figures 3B), indicating that *pelo* deficiency did not affect Dicer-2 pathway. DCV-induced JAK-STAT activation can be measured by the expression of its target gene *vir-1* [15]. The induction of *vir-1* by DCV in *pelo^−/−^* flies was not enhanced but even reduced when compared with wild-type flies (Figure 3C). However, when *pelo* knockdown S2 cells were used, we did not detect any effect of *pelo* deficiency on DCV induced expression of *vir-1* (Figure S4). While we do not know why there is difference between fly and S2 cells in JAK-STAT activation, these data still excluded the possibility that DCV resistance in *pelo* deficient flies or cells is due to an enhancement of JAK-STAT activation.

### *pelo* deficiency selectively inhibits expression of DCV capsid proteins

The process of viral replication includes attachment, penetration, uncoating, biosynthesis of viral nucleic acids and proteins, assembly, and release [46]. To understand how *pelo* deficiency inhibits DCV replication, we ought to determine which step of DCV replication is affected by *pelo*. Since DCV is a positive-sense RNA virus, inhibition of protein synthesis by cycloheximide should block viral replication but not affect DCV entry. We therefore infected S2 cells with DCV in the presence of cycloheximide and analyzed the internalized DCV genomic RNA by qRT-PCR. The levels of DCV RNA in the control and *pelo* knockdown S2 cells were the same (Figure 3D), indicating that loss of *pelo* did not affect the efficiency of DCV entry.

Then we wanted to analyze the biosynthesis of viral nucleic acids and proteins. We measured the RNA level of DCV by qRT-PCR and labeled DCV-infected S2 cells with ^35^S-Met at different time points post-infection. We found that at 6 hour post-infection, there is no significant difference in viral RNA amounts between control and *pelo* knockdown S2 cells but the newly synthesized viral protein is less in *pelo* knockdown cells (Figure S5), suggesting that *pelo* plays a promoting role in viral protein synthesis.

**Figure 4:**
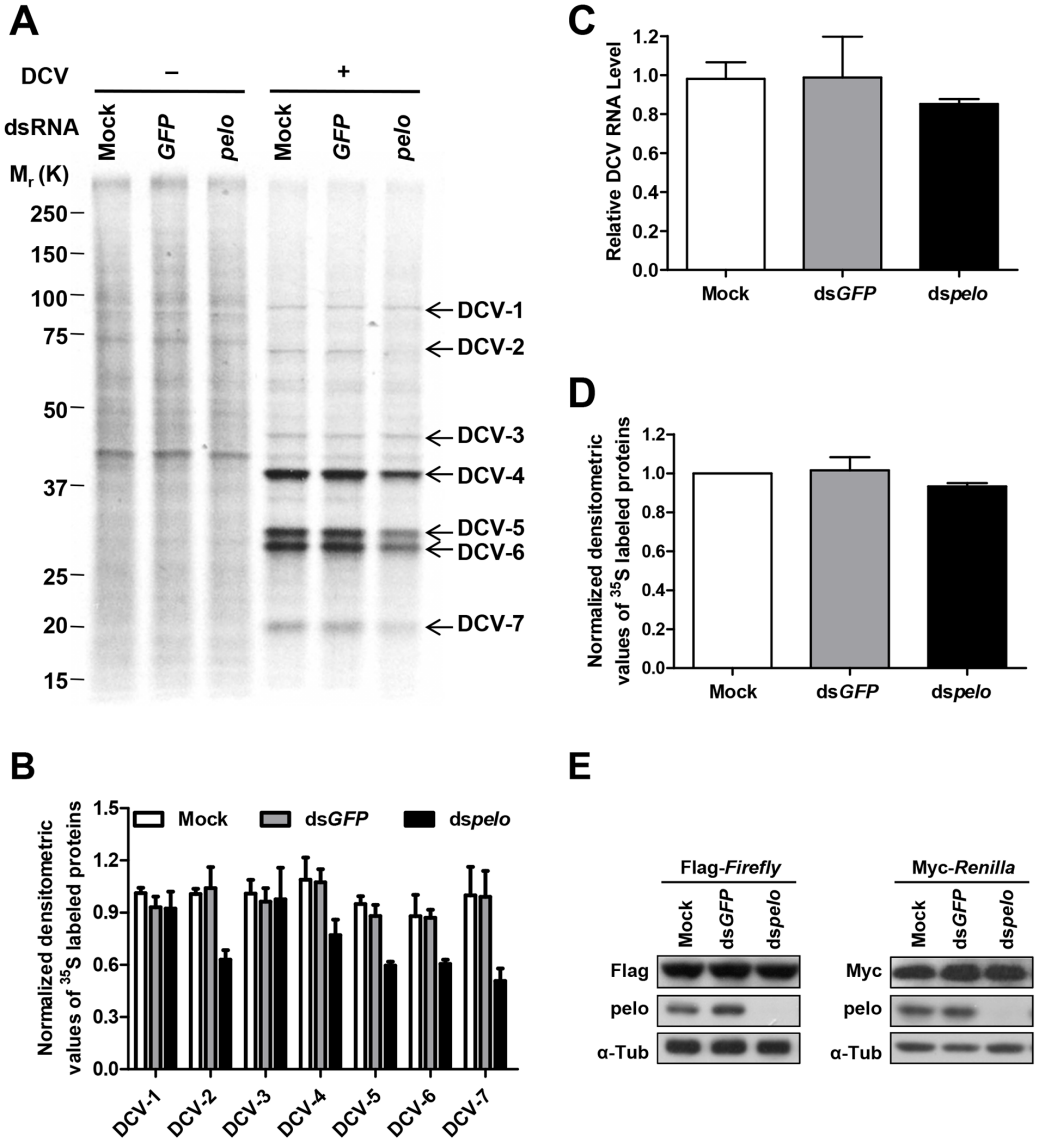
*pelo* regulates the syntheses of some specific DCV proteins. (**A**) Cells pretreated with the indicated dsRNAs were either uninfected (−) or infected (+) with DCV (MOI = 10) for 6 hours and then labeled with ^35^S-Met for 30 min. Labeled proteins were analyzed by Bis-Tris SDS-PAGE followed by autoradiography. The proteins that appeared after DCV infection are indicated by arrows and named DCV-1 to DCV-7. One ^35^S-Met labeled host cell protein between 37 and 50 KDa was used as loading control for protein analysis. (**B**) The quantities of DCV-1 to DCV-7 were determined by densitometry and normalized to mock control. The value of mock control was set at 1, and all other samples were normalized to it. (**C**) qRT-PCR analysis of DCV genomic RNA in different cells after 6 hours of DCV infection. Results were normalized to mock control and represent the mean ± SD of triplicates. (**D**) The quantities of metabolically labeled proteins (15–250 KD) were determined as decreased in **B**. (**E**) Immunoblot analysis of Flag-Firefly or Myc-Renilla expression in cells pretreated with indicated dsRNAs.

DCV contains two open reading frames (ORFs). ORF1 encodes a ∼200 kDa polyprotein that includes the domains of helicase, protease, and RNA-dependent RNA polymerase. ORF2 encodes a ∼100 kDa polyprotein which is subsequently cleaved into the capsid proteins [47]. There is no subgenomic RNA transcription during the DCV lifecycle and translation of both ORFs proceeds from the genomic RNA [48]. To further analyze DCV protein synthesis, we labeled DCV-infected S2 cells with ^35^S-Met at 6 hours post-infection for 30 min. 7 proteins were detected in DCV-infected cells, but not in non-infected cells, and were named DCV-1 to 7 (Figure 4A). In order to make sure that these proteins were all viral proteins and not the host proteins induced by viral infection, we used actinomycin D, which can inhibit host transcription and thus inhibit protein synthesis, with the exception of DCV proteins. As expected, actinomycin D treatment greatly reduced the amount of host proteins but had no effect on DCV-1 to 7 (Figure S6A), demonstrating that all these proteins were encoded by viral RNA. *pelo* deficiency reduced the expression of DCV-2, DCV-4, DCV-5, DCV-6, and DCV-7, but had very little to no effect on the expression of DCV-1 and DCV-3 (Figures 4A and 4B). Analysis of viral RNA in the control and *pelo* knockdown cells revealed no significant difference at 6 hours post-infection (Figure 4C). These results suggested that *pelo* regulates the expression of some but not all DCV proteins at the translational level.

The molecular weights of DCV-4 to 6 correspond to those of DCV capsid proteins [41]. More convincingly, antibodies against DCV capsid proteins recognized protein bands corresponding to the sizes of DCV-4 to 7 (Figure 2C and data not shown). The levels of these proteins are much higher than the levels of DCV-1 to 3, supporting the prediction that DCV-4 to 7 are in fact capsid proteins. To determine the identities of these proteins, we cut their bands from the Coomassie blue-stained SDS-PAGE gel and did mass spectrometry analysis (Table S1). Identified peptides of DCV-1 were located in the 975–1759 aa of ORF1, including the protease and RdRp domains. Peptides of DCV-3 were in the region of 297–679 aa, which includes the helicase domain in ORF1. Peptides of DCV-4 were in the 291–623 aa of ORF2, which corresponds to VP0. Because we could not separate DCV-5 and 6 well, the identified peptides of these two protein bands were almost the same. The peptides were distributed along the whole sequence of ORF2, consistent with the prediction that they are the mixture of VP1, VP2, and VP3. Peptides of DCV-7 were in the 647–850 aa of ORF2, indicating that DCV-7 is also encoded by ORF2. Interestingly, we could not find the corresponding band of DCV-2 in the Coomassie blue-stained gel despite its ^35^S-Met labeling level being equal to that of DCV-1 and DCV-3. We then analyzed the stability of the DCV proteins by a pulse-chase experiment and found that DCV-2, but not the other DCV proteins, had very short half-life (Figures S6B and S6C). *pelo* knockdown did not affect the turnover of these DCV proteins. The short half-life of DCV-2 explains why it is ^35^S-visible but Coomassie-invisible, because quick turnover make it rich in ^35^S-Met labeling (newly synthesized proteins) but its total amount is low. Because DCV-2 has almost equal ^35^S-Met labeling intensity as DCV-1 and DCV-3 but its half-life is much shorter than theirs, the instantaneous expression of DCV-2 should be higher than DCV-1 and DCV-3. Thus, DCV-2 may not be an ORF1 protein, but an ORF2 protein. Based on its size, DCV-2 cannot be the entire polyprotein of ORF2 or VP0. Since inhibition of either proteasomes or lysosomes by MG132 or chloroquine, respectively, could not increase the half-life of DCV-2 (data not shown), we predict that DCV-2 is an unprocessed precursor or intermediate product of capsids.

*pelo* deficiency apparently does not influence the synthesis of most, if not all kinds of cellular proteins in S2 cells based on the ^35^S-Met labeling experiments (Figures 4A, 4D and S6). The levels of proteins expressed by transiently transfected plasmids in the control and *pelo* knockdown cells were the same or almost the same (Figure 4E). Taken together, these data suggested that *pelo* is selectively involved in the expression of some DCV proteins such as capsids.

### *pelo* does not target IRES or the termination sequence of ORF2

**Figure 5:**
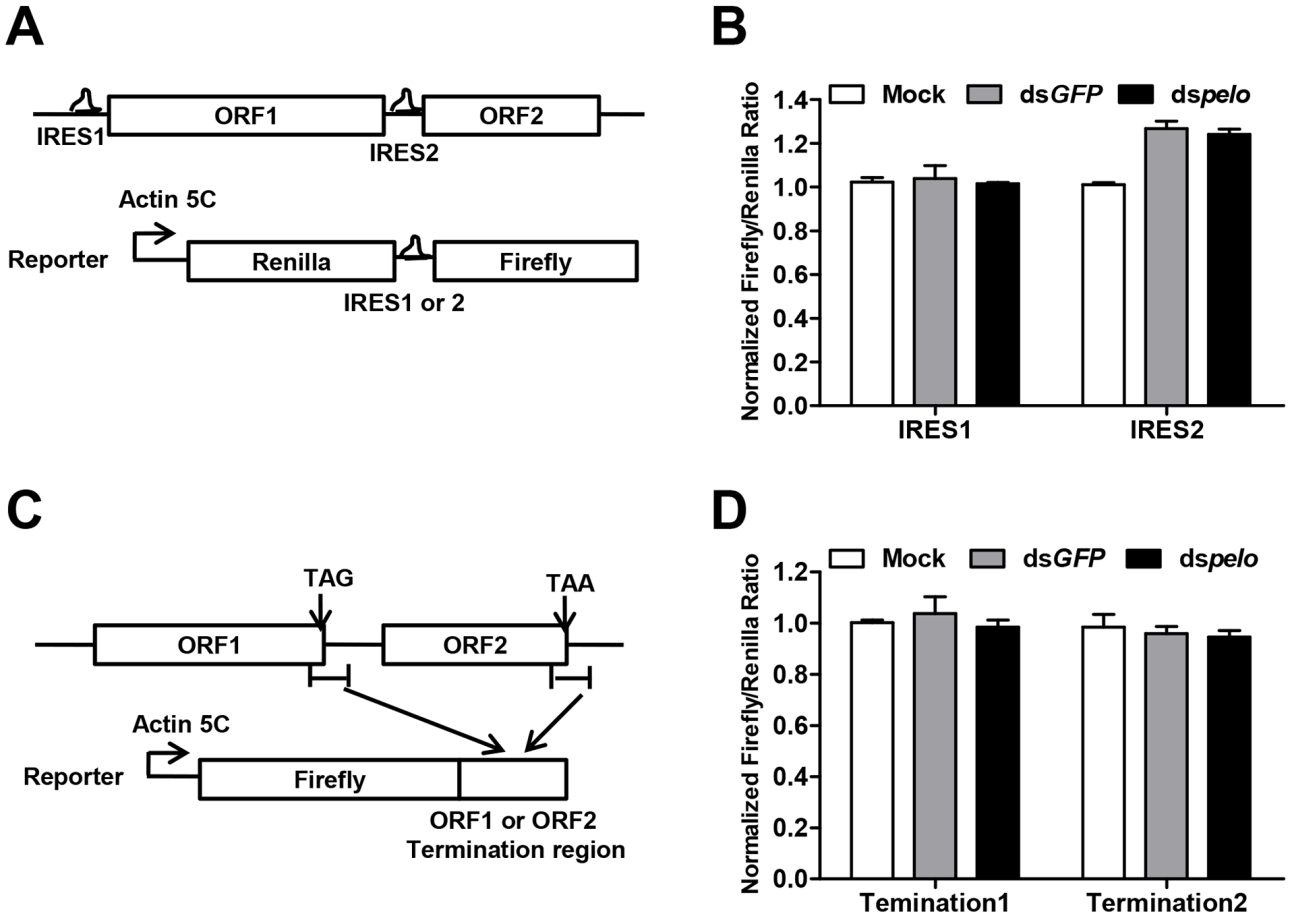
*pelo* does not regulate IRES or the termination sequence of ORF2 of DCV. (**A**) Schematic diagram of the bicistronic reporter constructs. Translation from 5′cap (5C) is quantified by Renilla luciferase, while translation from the DCV IRES1 or IRES2 is measured by Firefly luciferase. (**B**) Luciferase activities were assayed at 36 hours after transfection of DCV IRES1 or IRES2 reporter plasmid. Ratios of Firefly to Renilla luciferase activity are shown after they were normalized to untreated cells (Mock), which was set at 1. Data are the mean ± SD of triplicates. (**C**) Schematic diagram of the Firefly luciferase reporter constructs. Termination region of DCV ORF1 or ORF2 (between ∼100 bp upstream and ∼200 bp downstream of stop codon) was fused to the c-terminal of Firefly luciferase. (**D**) Cells pretreated with indicated dsRNAs were transfected with each of the Firefly luciferase reporters shown in (**C**) together with a constitutively Renilla luciferase-expressing vector. Luciferase activities were assayed as described in (**B**).

Since capsid proteins of DCV are encoded by ORF2, *pelo* may regulate the translation of DCV ORF2. The translation initiations of DCV ORFs are mediated by two different internal ribosomal entry sites (IRES). We constructed bicistronic luciferase reporters containing either the DCV IRES1 or IRES2 (Figure 5A), and neither of these two IRES-dependent translations was influenced by the depletion of *pelo* (Figure 5B). Because the structure of *pelo* is similar to eRF1 and *pelo* has functions in promoting ribosomal subunit dissociation, we tested whether the termination sequence of ORF2 is regulated by *pelo*. We fused termination regions of ORF1 or ORF2 to *Firefly* luciferase reporters (Figure 5C), and found that the expression of both reporters was not influenced by *pelo* knockdown (Figure 5D). Our reporter studies suggested that *pelo* does not function in the regulation of either IRES dependent translational initiation of ORF2 or termination of ORF2.

### The increased non-functional 80S monoribosomes in *pelo* deficient cells may limit ribosome availability and contribute to the inhibition of DCV protein synthesis

**Figure 6:**
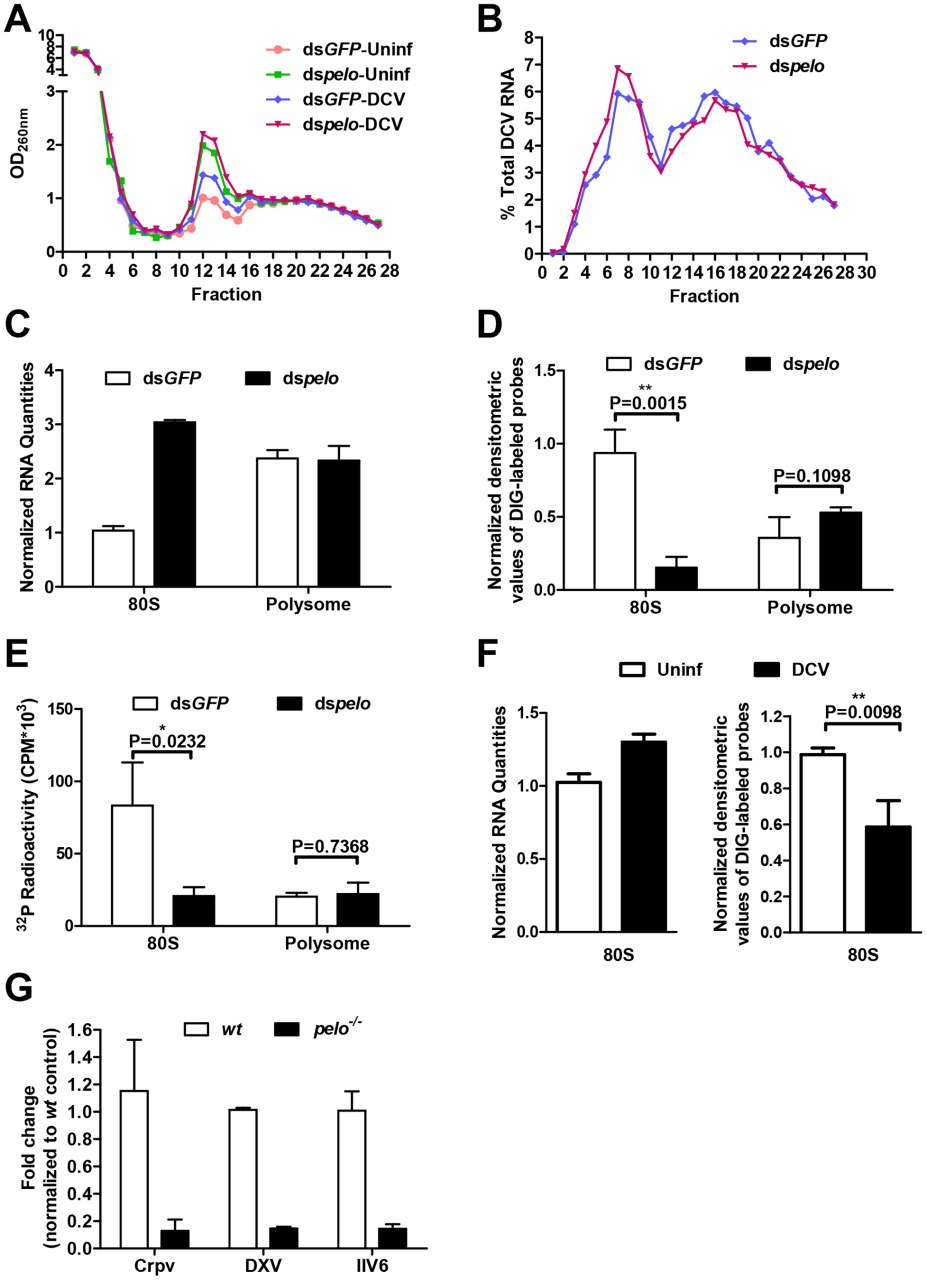
The increased non-functional 80S monoribosomes in *pelo*-deficient cells may limit free ribosome availability for high level viral protein synthesis. (**A**) Cells pretreated with the indicated dsRNAs were either uninfected (Uninf) or infected with DCV for 6 hours (MOI = 10) followed by polysome profile analysis. (**B**) qRT-PCR analysis of DCV RNA in individual fractions of sucrose gradients. The distribution of DCV RNA among the fractions is shown as a percentage of the total DCV RNA. (**C**) Total RNA of the 80S fraction and one of the polysome fractions were extracted. RNA quantities are shown as their relative ratios compared to that of 80S in ds*GFP*-treated cells. (**D**) Equal amounts of RNA from each sample were hybridized with digoxigenin (DIG)-labeled oligo (dT) probes and then applied to the nylon membrane. The membrane was probed with anti-DIG antibody and detected by chemiluminescence. The quantities of DIG-labeled probes were determined by densitometric analysis and normalized to the value of 80S in ds*GFP*-treated control cells. (**E**) Equal amounts of RNA from each sample were reverse transcribed with oligo (dT) in the presence of [α-^32^P] dTTP. The resulting cDNAs were recovered by using NucAway spin column. The radioactivity was measured with liquid scintillation counting. Results are the mean ± SD of triplicates. (**F**) Cells were uninfected (Uninf) or infected with DCV for 12 hours (MOI = 30) and then were followed by polysome profile analysis. Total RNA of 80S fractions of each sample were extracted and then analyzed as described in **C** and **D**. (**G**) pelo is generally involved in the replication of different viruses. wt and pelo^−/−^ flies were infected with Crpv, DXV, or IIV6 for 72 hours. RNA was extracted from Crpv and DXV infected flies, and DNA was isolated from IIV6 infected flies. qRT-PCR or qPCR was used to analyze the amounts of these three different viruses. Results were normalized to wt and shown as the relative values. Data are the mean ± SD of triplicates.

Polysome profile analysis is frequently used to monitor the efficiency of translation [49]. We used it to examine whether there was any difference in the efficiency of DCV protein synthesis between control and *pelo* knockdown S2 cells. Cells were mock infected or infected with DCV for 6 hours. The 6 hours was chosen because at this time point the difference in DCV protein, but not DCV RNA, begins to appear between wild-type and *pelo* deficient cells (Figure S5). Cell lysates were resolved on a 10–50% continuous sucrose gradient (Figure 6A). Knockdown of *pelo* increased the amount of 80S monoribosomes, which is consistent with the result obtained by studying Dom34 deletion in yeast [37]. However, the distribution of DCV RNAs in ribosome profiling fractions is about the same in wild-type and *pelo* knockdown cells (Figure 6B). DCV infection also leads to increase of 80S monoribosomes in wild-type S2 cells over the time of infection (Figures 6A and S7). DCV infection does not affect the level of 80S monoribosome in *pelo* deficient cells much (Figure 6A), due to the already high level of 80S monoribosome and ineffective replication of DCV in *pelo* deficient S2 cells.

The 80S monoribosomes could associate with mRNA and also could be free. We extracted the total RNAs from 80S and polysome fractions prepared from non-DCV infected cells. The amount of RNA in the 80S fraction from the *pelo*-deficient cells was about three times of that from the control cells, while the quantities of RNA in the polysome fraction were about the same in both the control and *pelo* knockdown cells (Figure 6C). Because rRNA is the major component of the total RNA extracted from these fractions, this data confirms that *pelo*-deficient cells have more 80S monoribosomes. We then analyzed the amount of mRNA associated with 80S monoribosomes and polysomes. The samples with equal amounts of total RNA, which indicated that the RNA samples were extracted from an equal number of ribosomes, were hybridized with digoxigenin (DIG)-labeled oligo (dT) probes to detect mRNA. DIG was detected with alkaline phosphatase labeled anti-DIG antibody by chemiluminescence (Figure 6D). The amount of Poly (A) RNA in the 80S fraction of the *pelo*-deficient cells was less than that of the control cells, whereas poly (A) RNA amounts in the polysome fraction were not affected by *pelo* knockdown. The same result was obtained by reverse transcribing total RNA with oligo (dT) in the presence of [α-^32^P] dTTP (Figure 6E). The level of incorporated [α-^32^P] dTTP in cDNAs should correlate with the level of mRNA. Based on these data, we concluded that the increased portion of 80S monoribosomes in *pelo*-deficient S2 cells was primarily contributed to by mRNA free ribosomes. By using the same approach, we determined that DCV infection-induced 80S monoribosomes in wild-type S2 cells are also mainly poly (A) RNA free ribosomes (Figure 6F).

Since Dom34 forms a complex with HBS1 to function [29], [50], we knocked down *HBS1* in S2 cells and found that DCV replication was indeed inhibited (Figure S8). It has been well established recently that Dom34-HBS1 plays an essential role in the quality control of protein translation by dissociation of stalled ribosomes at the 3′ end of aberrant mRNAs. The increase of 80S monoribosomes in *pelo*-deficient cells is most likely to be a result of those stalled ribosomes being processed to monomers. The increase in non-translating 80S ribosomes in DCV infected cells suggests that high level DCV protein synthesis produces more incidents of stalled ribosomes. Inefficient recycling of stalled 80S ribosomes in DCV RNA should result in the synthesis of truncated and mutated viral proteins and also could reduce the translation efficiency of viral RNA. Impairment of the dissociation of non-translating 80S ribosomes should reduce the availability of ribosomes for protein translation. It is believed that the relationship between free ribosome concentration and the rate of peptide formation follows the Michaelis-Menten equation [51] (Figure S9). Based on the Michaelis-Menten equation, the reduction of free ribosome concentration has a more inhibitory effect on the production of quickly synthesized proteins than on the production of slowly formed proteins (Figure S9). In the case of DCV, a single viral RNA encodes capsid proteins DCV-4 to 7 and non-capsid proteins DCV-1 and 3. Since a large amount of capsid proteins is required for efficient DCV replication, reduced free ribosomes in *pelo* knockdown cells are likely to be responsible for the phenomenon in which *pelo* knockdown had much more of an effect on the protein synthesis of DCV-4 to 7 than on that of DCV-1 and 3 (Figure 4).

High speed synthesis of capsid proteins is required for the replication of many different viruses. In order to further evaluate the notion that the availability of free ribosomes in *pelo*-deficient flies limits viral capsid protein synthesis, we examined whether *pelo* deficiency can influence the replication of three other viruses, single-stranded RNA virus *Cricket Paralysis Virus* (Crpv), double-stranded RNA virus *Drosophila X virus* (DXV), and a large dsDNA virus *invertebrate iridescent virus 6* (IIV6). We used qRT-PCR as described by published studies [15], [17], [52] to measure the corresponding RNA or DNA levels of each virus in the infected flies, and found that the replications of these viruses were all slower in *pelo* ^−/−^ flies (Figure 6G). We also analyzed Crpv, DXV, and IIV6 at different time points after infection in wild-type and *pelo* ^−/−^ flies and measured viral titers in flies at 3 days post-infection (Figure S10). The data support the conclusion that the replications of these three different viruses are suppressed in *pelo* ^−/−^ flies. The *pelo* deficiency selectively resists viral infection as *pelo* ^−/−^ flies are not resistant but even more sensitive to bacterial infection (data not shown). These data support the idea that the defect in the recycling of stalled ribosomes in *pelo*-deficient cells limits viral replication and implies that *pelo* deficiency mediates a general antiviral activity.

## Discussion

Through a forward genetic screen for *Drosophila* mutants with increased or decreased susceptibility to DCV-induced death, we have identified a virus-resistant line in which the *pelo* gene was mutated. We further demonstrated, by using this and other *pelo*-deficient *Drosophila* lines, and by rescuing *pelo* expression in *pelo* ^−/−^ flies, that *pelo* deficiency led to resistance to DCV infection (Figure 1). The *pelo* deficiency mediated DCV resistance can be phenotypically copied in S2 cells (Figure 2). Our mechanistic studies revealed that *pelo* deletion led to inefficient DCV replication (Figure 2). pelo protein appears to be involved in the high efficiency protein translation of DCV capsid proteins (Figure 4). Our data suggest that the role of *pelo* in the dissociation of 80S ribosomes links to *pelo* deficiency mediated resistance to DCV infection. Huge amounts of capsid protein synthesis in DCV infected cells could increase errors in protein translation. The increase in 80S ribosomes that were not associated with poly (A) mRNA in DCV infected cells suggests an increase of stalled ribosomes (Figure 6). Deletion of *pelo* impairs the recycling of the stalled ribosomes, which reduces the availability of free ribosomes and thus could be a limiting factor of DCV replication in *pelo*-deficient cells. Indeed, the effect of *pelo* deficiency on high speed translation of capsids is much greater than that on slowly translated DCV-1 and DCV-3 proteins from the same RNA (Figure 4), consistent with the kinetic prediction that a more quickly synthesized protein is limited more effectively than a more slowly translated protein by available free ribosome concentration (Figure S9). In addition, inefficient removal of stalled ribosomes from a given DCV RNA could also eliminate this DCV RNA from translation. It could create truncated or mutated DCV capsid proteins that might interfere with DCV assembling and further inhibit DCV replication. All these mechanistic predictions point to a general antiviral role of *pelo* deficiency. We have further confirmed that *pelo* ^−/−^ fly is indeed resistant to a broad panel of different viruses including a single-stranded RNA virus, a double-stranded RNA virus, and a DNA virus.

*pelo* may function beyond recycling stalled 80S ribosomes as a recent study showed that the formation of 80S-like complexes is an intermediate step in the process of 40S ribosome maturation, and *pelo* is involved in the dissociation of this 80S-like complex during the maturation of the 40S ribosome [53]. Regardless of whether *pelo* dissociates stalled 80S ribosomes or the 80S-like complex, it affects the supply of free ribosomes. pelo is known to form a complex with HBS1 to dissociate ribosomes that are vacant or stalled at the aberrant mRNA [27], [28], [29], [31], [32], [33], [54], which is supported by our data that *HBS1* knockdown also inhibits DCV replication. *pelo* knockout is lethal in mice but not in yeast and *Drosophila* as we presented here [36], [38], [40], suggesting that *pelo* is not required for cell survival but is instead required for mouse development during the embryonic stage. *pelo* should also be important for the expression of certain genes since its knockout leads to male-sterility in flies (refs [38], [39]and data not shown). The selective inhibition of DCV capsid protein expression by *pelo* knockdown strongly suggests that *pelo* is not generally involved in the protein translation of all proteins. At present we know little about the identity of the cellular proteins whose translations are regulated by *pelo*. The current study revealed that at least some of the quickly synthesized proteins are subject to control by *pelo*. The regulation of the availability of free ribosomes should be at least part of the underlying mechanisms. The Michaelis-Menten equation-based prediction is certainly an over-simplified explanation since cellular processes are compartmentalized and the local concentration of free ribosomes in the synthesis site of a given protein is important. If certain protein synthesis sites are more dependent on recycled free ribosomes, *pelo* should have more influence on the synthesis of these proteins than those synthesized in other locations. Unfortunately, current technologies are unable to address this question.

It is known that multiple antiviral pathways including RNAi and antiviral gene expression are present in *Drosophila* [8]. Our data excluded the possibility that *pelo* deficiency mediated inhibition of viral replication resulted from the activation of these antiviral mechanisms (Figure 3). Without exception, viruses use cellular translational machinery to synthesize their proteins. *pelo* should belong to those proteins that are hijacked by infected virus to highly efficiently translate viral proteins. Because *pelo* is not required for cell survival, it is dispensable for protein synthesis of most cellular proteins. The general suppressive effect on virus replication imposed by *pelo* deficiency suggests that *pelo* is involved in a unique part of translational machinery used by viruses. Since viral replication requires high efficiency synthesis of a large amount of viral capsids, it is possible that viruses are able to utilize special cellular mechanisms for quickly synthesizing a large amount of proteins. If it is the case, *pelo* is involved in this special mechanism.

There is no doubt that viral recognition mechanisms in cells and the subsequent activation of antiviral responses are essential for host defense against viral infection. However, the cellular mechanisms that are preferentially hijacked by viruses are also important parts of host-virus interaction. Given the general antiviral effect of *pelo* deficiency, the factors that are dispensable for normal cell function but are involved in viral protein synthesis should draw more attention in our studies of host response to viral infection. Our work presented here identifies a new host factor that is essential for effective viral replication. Since *pelo* is a highly conserved protein, the function of *pelo* revealed in this study is very likely to be conserved in mammals as well. The discovery of the general antiviral activity by *pelo* deficiency may provide a new therapeutic target for broad-spectrum antiviral therapy.

## Materials and Methods

### Fly strains, screening, and viral infections

Flies were raised on a standard yeast-cornmeal-agar medium and all experiments were performed at 25°C. A large-scale P-element mutagenesis was performed by mobilizing a P {Mae-UAS.6.11} transposon (Bloomington stock #3025) from the X chromosome to random autosomal sites with the transposase P {Δ2-3} 99B (Bloomington stock #3664) by standard methodology. The insertion lines were infected with DCV and selected for more resistant or sensitive lines. We screened about 100 lines and selected a resistant line, *R32*. Identification of the P-element insertion site in *R32* was performed by inverse PCR.

The *pelo^−/−^* line was generated by imprecise excision of the P-element from the line *R32* and contained a deletion of about 1.1 kb including exon 1 and exon 2. A precise P-element excision line was used as a wild-type (*wt*) control. All other stocks were obtained from the *Drosophila* Bloomington stock center unless indicated otherwise.

For viral infection, 2–4 days old flies of the stated genotype were infected by septic injury with the indicated virus as described [55]. Briefly, the thorax of the fly was pricked with a thin needle previously dipped into the viral solution (DCV, 5×10^11^ TCID_50_/ml; Crpv, 4×10^7^ TCID_50_/ml; DXV, 6×10^7^ TCID_50_/ml; IIV6, 7×10^10^ TCID_50_/ml) and only the very tip of the needle is inserted into the fly. The infected flies were monitored for mortality or collected at indicated time points for different assays. Viral titers were determined in S2 cell culture and calculated according to the end-point method of Reed and Muench [56].

### Cells, RNAi and transfection

*Drosophila* S2 cells (ATCC) were cultured at 25°C in SF900-II SFM medium (Gibco). dsRNA targeting *pelo* (nucleotides 304 bp–938 bp), *HBS1* (nucleotides 706–2119 bp), *Firefly* (nucleotides 1144–1650 bp), and *GFP* (nucleotides 65–559 bp) were generated from T7-promoter-flanked PCR products by in vitro transcription using T7 transcription kit (Promega). RNAi treatments were performed as previously described [57]. In brief, S2 cells were incubated with SF900-II SFM medium containing 10 ug/ml dsRNA and then were replenished with fresh dsRNA-containing medium daily for 6 days.

For transfection, S2 cells were incubated in Schneider's medium (Lonza) supplemented with 10% FBS, and transfections were performed by the Calcium Phosphate precipitation method. After 12 hours, the medium was removed and replaced with SF900-II SFM medium. Luciferase expression was assayed using the Dual-Luciferase Reporter Assay System (Promega) and quantitated on the luminometer.

### Quantitative RT-PCR

Total RNA was extracted from adult flies or S2 cells with RNAiso Plus (Takara) according to the manufacturer's instructions. cDNA was prepared with M-MLV reverse transcriptase and oligo-dT primers. Quantitative RT-PCR was performed using SYBR Green reagent along with gene-specific primers. All the results were analyzed by relative quantification, by normalizing to the *Rp49* RNA level. Primer sequences are available on request.

### Western blots

Cells or flies were lysed in Laemmli sample buffer and then boiled. Samples were separated by SDS-PAGE and blotted onto PVDF membrane. The blots were probed with indicated antibodies and detected by Chemiluminescent HRP Substrate (Millipore). Antibodies were obtained from the following sources: anti-α-tubulin (BGI), anti-Flag (Abmart), anti-Myc (Santa Cruz), and polyclonal antibodies to pelo and DCV were prepared in rabbits against bacterially expressed full-length pelo protein or purified DCV particles respectively.

### Metabolic labeling

Cells were washed three times with PBS and then starved with methionine-free culture medium for 15 min. After methionine starvation, cells were incubated with 0.2 mCi/ml ^35^S-Methione in methionine-free medium for 30 min, washed three times with PBS and lysed with Laemmli sample buffer. Labeled proteins were resolved by Bis-Tris SDS-PAGE and then fixed. The dried gels were exposed to film. For protein stability analysis, cells were labeled with ^35^S-Met for 30 min, washed three times with PBS and then lysed (time point 0) or chased for the indicated time with excess cold methionine.

### Polysome analysis

Cells were incubated with 100 µg/ml cycloheximide for 10 min and then washed twice with cold PBS containing cycloheximide (100 µg/ml). 5×10^8^ cells were lysed with 1 ml polysome lysis buffer (10 mM HEPES-KOH pH 7.4, 5 mM MgCl2, 150 mM KCl, 0.5% NP-40, 0.5 mM DTT, 100 µg/ml cycloheximide, 100 U/ml RNAsin RNase inhibitor [Promega], and EDTA-free protease inhibitor cocktail Complete [Roche]). Cell debris was removed by centrifuging for 10 min at 16,000 g. The cytoplasmic supernatant was layered onto 10 ml of 10–50% continuous sucrose gradient and centrifuged at 4°C for 2 h at 36,000 rpm in a Beckman SW41 rotor. Then 0.4-ml fractions were collected from the top of the gradient and the polysome profile was monitored by RNA absorbance at 260 nm.

RNA of each fraction was extracted by using RNAiso Plus (Takara) and the distribution of DCV RNA was measured by quantitative RT-PCR. For the slot blot assay, RNA was incubated with digoxigenin (DIG)-labeled oligo (dT) probes. After hybridization, samples were applied to the nylon membrane by using a Bio-Dot apparatus (Bio-rad) and fixed by UV crosslinking. Following the washing and blocking steps, the membrane was probed with anti-DIG antibody that was coupled to alkaline phosphatase (Roche), and then detected by incubating with the chemiluminescent substrate CDP-star and exposing the blot to X-ray film. The quantities of DIG-labeled probes were determined by densitometric analysis. For radioactive assay, equal amounts of RNA from each sample were reverse transcribed with oligo (dT) in the presence of [α-^32^P] dTTP. The resulting cDNAs were recovered by using NucAway spin column (Ambion) while removing the salts and unincorporated [α-^32^P] dTTP. The radioactivity was measured with liquid scintillation counting (Beckman).

### Statistical analysis

Statistical analysis was performed using the unpaired two-tailed Student's t-test with the Prism GraphPad software. *P*<0.05 was considered significantly different.

### Accession numbers

#### NCBI gene ID numbers for *Drosophila melanogaster* genes mentioned in the text

*pelo* (34286); *HBS1* (117365); *Pka-C1* (34284); *hoip* (44173); *CG31710* (318907); *vir-1* (34652); *vago* (32040).

#### Genebank accession numbers for the genome sequences of the viruses

*Drosophila C virus* (DCV), NC_001834; *Drosophila x virus* (DXV), NC_004177, NC_004169; *Cricket paralysis virus* (Crpv), NC_003924; *Invertebrate iridescent virus 6* (IIV6), NC_003038.

## Supporting Information

Figure S1 **Screen for Drosophila mutants with increased or decreased susceptibility to DCV-induced death.** 2–4 days old flies were injected with DCV and then monitored for mortality. y w was used as a genetic background control. 60 flies of each line were used. About 100 mutant fly lines were screened and ten lines were shown.
(TIF)

Figure S2 **Measuring the** ***Wolbachia*** **infection status of the fly lines used.** (**A**) PCR amplification with primers for the Wolbachia specific genes wsp and wspB on DNA extracts of indicated fly lines. PCR amplification of mt 12S rRNA was used as a DNA extraction control. (**B**) The amounts of Wolbachia DNA in indicated fly lines were measured by qRCR. Results were normalized to y w and shown as the relative values. Data are the mean ± SD of triplicates.
(TIF)

Figure S3 **Growth curve of cells pretreated with indicated dsRNAs.** Cells pretreated with the indicated dsRNAs for 6 days were seeded at a density of 10^6^ cells/ml (day 0) and then counted every day. Data are the mean ± SD of triplicates.
(TIF)

Figure S4 **The expressions of JAK-STAT target gene after DCV infection in S2 cells.** S2 cells were untreated (Mock) or treated with the indicated dsRNAs for 6 days and then infected with DCV (MOI = 0.1). The expressions of *vir-1* (**A**) and the accumulation of DCV RNA (**B**) were analyzed by qRT-PCR. Data are the mean ± SD of triplicates.
(TIF)

Figure S5 **The biosynthesis of viral nucleic acids and proteins during the course of DCV infection.** (**A**) Cells pretreated with the indicated dsRNAs were infected with DCV (MOI = 10) and then collected at different time points. The accumulation of DCV RNA was measured by qRT-PCR. Data are the mean ± SD of triplicates. (**B**) Cells were labeled with ^35^S-Met for 30 min at different time points post-infection. Labeled proteins were analyzed by Bis-Tris SDS-PAGE followed by autoradiography. One ^35^S-Met labeled host cell protein between 37 and 50 KDa was used as loading control for protein analysis.
(TIF)

Figure S6 **DCV-1 to 7 are viral proteins and DCV-2 has high rate of turnover.** (**A**) Cells pretreated with the indicated dsRNAs were either uninfected or infected with DCV (MOI = 10) for 2 hours and then actinomycin D was added to the medium to inhibit host mRNA transcription. 4 hours later, cells were labeled with ^35^S-Met for 30 min in the presence of actinomycin D or absence of actinomycin D. Labeled proteins were analyzed by Bis-Tris SDS-PAGE followed by autoradiography. (**B and C**) Uninfected (Uninf) cells or cells infected with DCV for 6 hours were labeled with ^35^S-Met for 30 min. Cells were washed three times with PBS and then lysed (time point 0) or chased for the indicated times with excess cold methionine. The stabilities of labeled proteins were detected by Bis-Tris SDS-PAGE, followed by autoradiography.
(TIF)

Figure S7 **Polysome analysis of DCV infected S2 cells.** Cells were infected with DCV (MOI = 10) and then collected at different time points for polysome profile analysis. Lysates were layered on a 10–50% sucrose gradient and centrifuge. 0.3-ml fractions were collected and the polysome profile was monitored by RNA absorbance at 260 nm. Note that there is an extra peak in DCV-infected cells at 12 h post-infection, which is most likely from the packaged viruses.
(TIF)

Figure S8 **Replication of DCV is slower in** ***HBS1*** **knockdown S2 cells.** (**A**) Cells pretreated with indicated dsRNAs were challenged with DCV and harvested at different time points post-infection. The accumulations of DCV capsid protein were measured by immunoblotting. (**B**) Knockdown efficiency of HBS1 was assessed using qRT-PCR. Results are the mean ± SD of triplicates.
(TIF)

Figure S9 **Mathematic calculation of the effect of ribosome concentration on different-speed synthesized proteins.** (**A**) Michaelis-Menten equation of formation of peptide. V_A_: rate of peptide A formation in wild-type cells. [S]: concentration of free ribosome in wild-type cells. V_maxA_: maximum rate of peptide formation. K_mA_, Michaelis constant of peptide A formation. (**B**) V′_A_: rate of peptide A formation in pelo^−/−^ cells. [S]′: concentration of free ribosome in pelo^−/−^ cells. ΔS: [S]-[S]′. (**C**) V_B_ and V_B_′: rate of B peptide formation in wild-type and pelo^−/−^ cells, respectively. K_mB_: Michaelis constant of peptide B formation. (**D**) The effect of decrease in free ribosome concentration [S] on the more quickly synthesized peptide A is greater than that on the more slowly synthesized peptide B.
(TIF)

Figure S10 ***pelo*** **deficiency inhibits the replication of different type viruses.** (**A–C**) wt and pelo^−/−^ flies were infected with virus and collected at the indicated time point. RNA was extracted from Crpv (**A**) and DXV (**B**) infected flies, and DNA was isolated from IIV6 (**C**) infected flies. qRT-PCR or qPCR was used to analyze the amounts of these three different viruses. (**D**) Flies were infected with indicated virus for 3 days. Three pools of ten flies were collected and homogenized. The viral titer in the homogenate was determined by end-point dilution. Data are the mean ± SD of triplicates.
(TIF)

Table S1 **Peptides identified in mass spectrometry.**
(XLSX)
