# Supplementary figures and images for: pelo Is Required for High Efficiency Viral Replication
Source: PLoS Pathog. 2014 Apr 10;10(4):e1004034. doi: 10.1371/journal.ppat.1004034 (PMC3983054; doi:10.1371/journal.ppat.1004034)

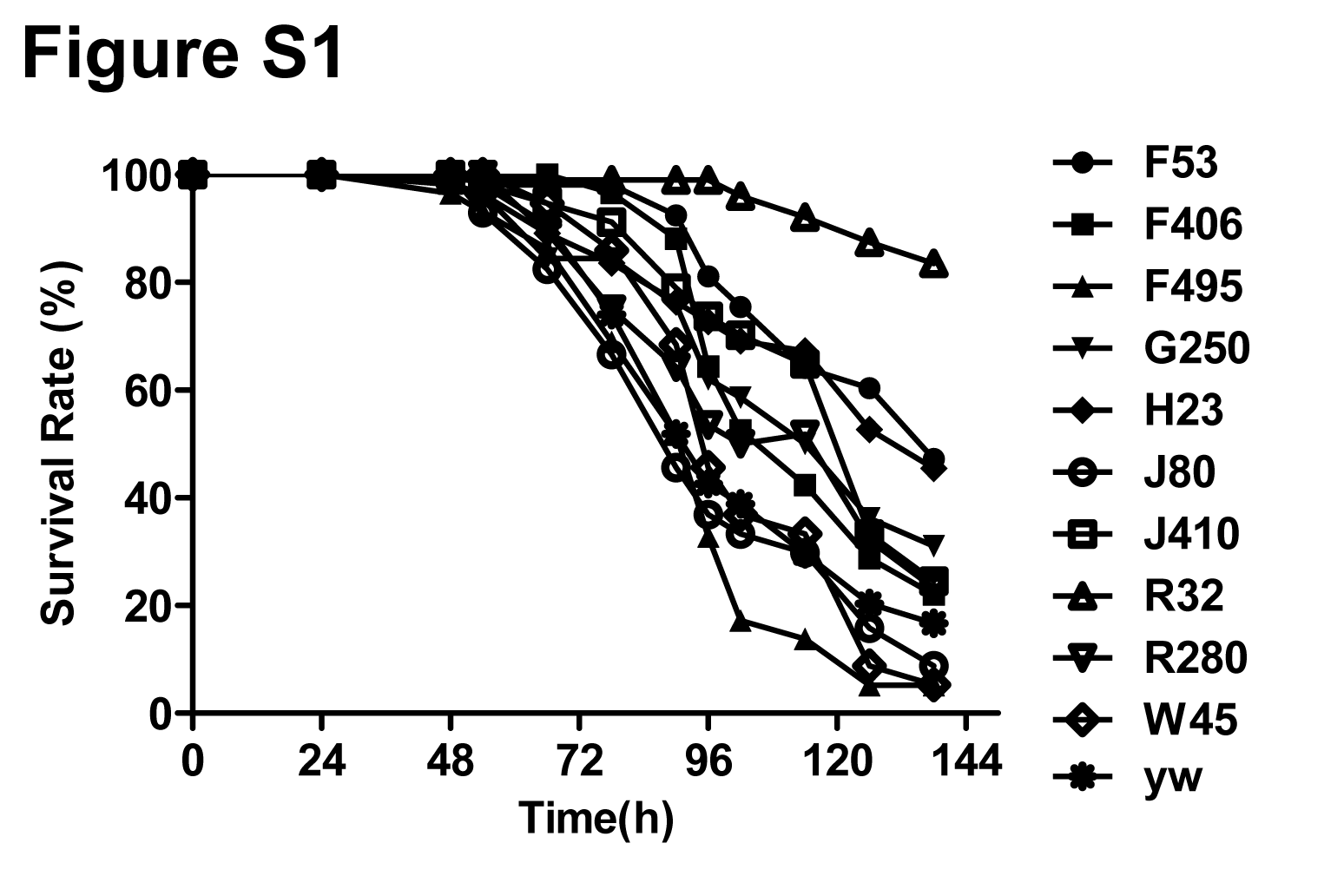

Supplement: Figure S1 — Screen for Drosophila mutants with increased or decreased susceptibility to DCV-induced death. 2–4 days old flies were injected with DCV and then monitored for mortality. y w was used as a genetic background control. 60 flies of each line were used. About 100 mutant fly lines were screened and ten lines were shown. (TIF) [file ppat.1004034.s001.tif]

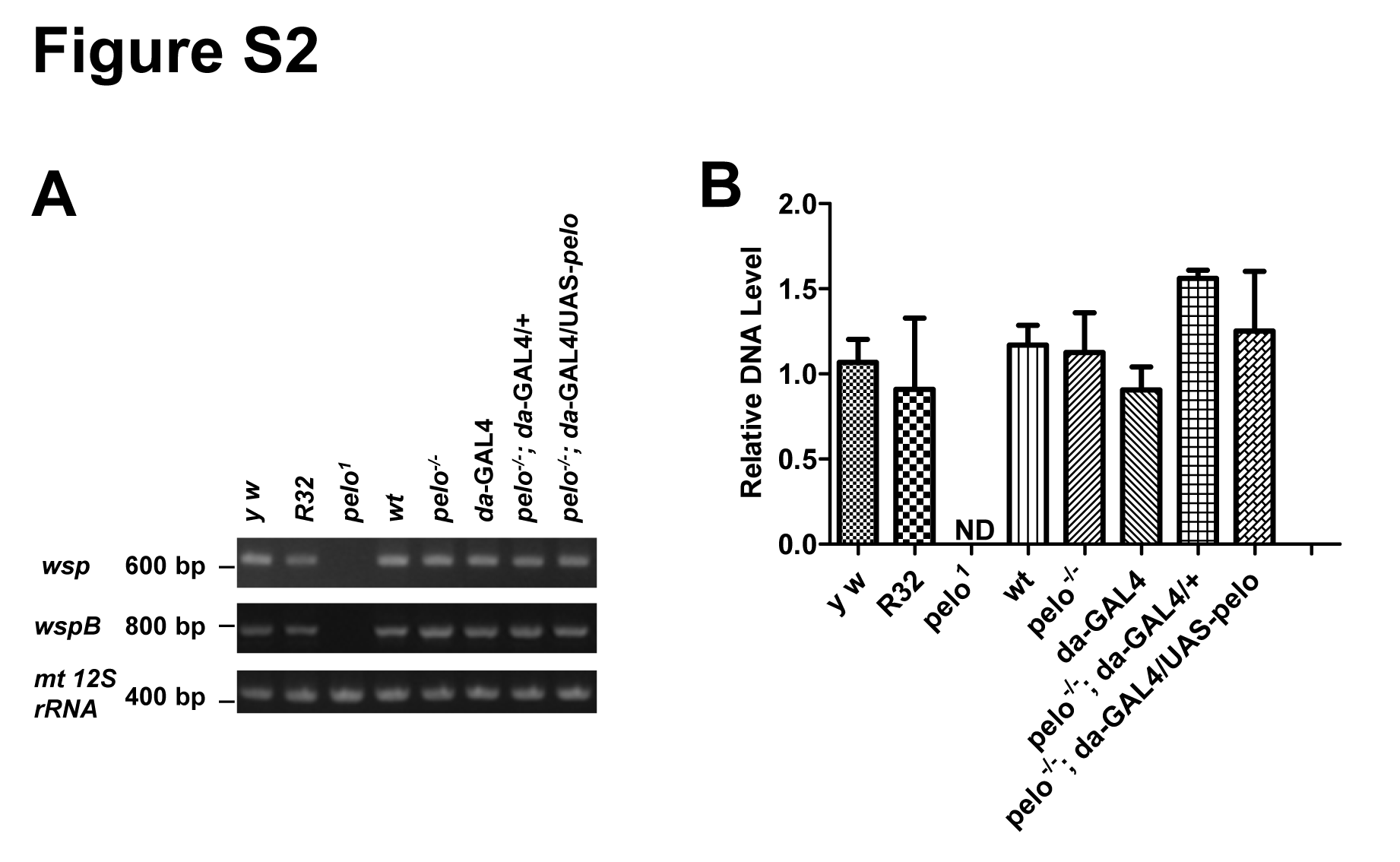

Supplement: Figure S2 — Measuring the Wolbachia infection status of the fly lines used. (A) PCR amplification with primers for the Wolbachia specific genes wsp and wspB on DNA extracts of indicated fly lines. PCR amplification of mt 12S rRNA was used as a DNA extraction control. (B) The amounts of Wolbachia DNA in indicated fly lines were measured by qRCR. Results were normalized to y w and shown as the relative values. Data are the mean ± SD of triplicates. (TIF) [file ppat.1004034.s002.tif]

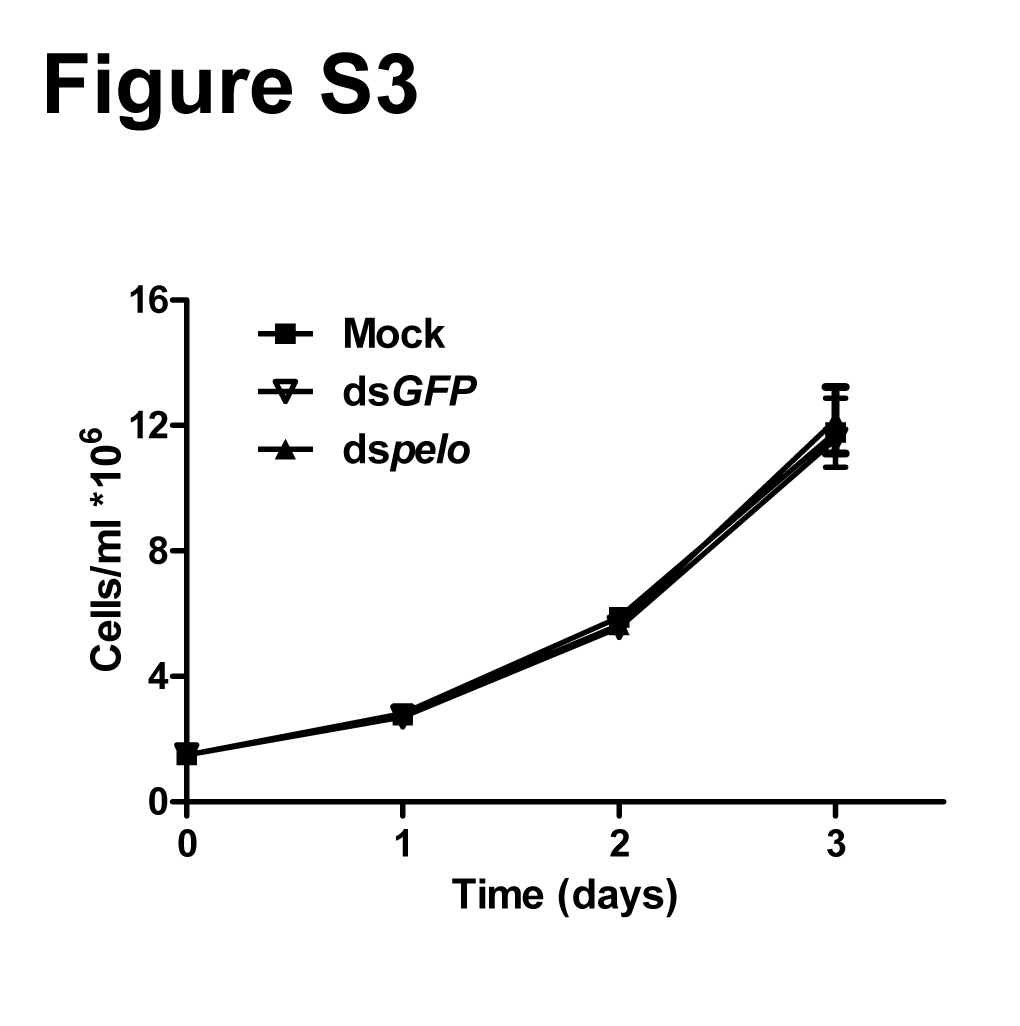

Supplement: Figure S3 — Growth curve of cells pretreated with indicated dsRNAs. Cells pretreated with the indicated dsRNAs for 6 days were seeded at a density of 106 cells/ml (day 0) and then counted every day. Data are the mean ± SD of triplicates. (TIF) [file ppat.1004034.s003.tif]

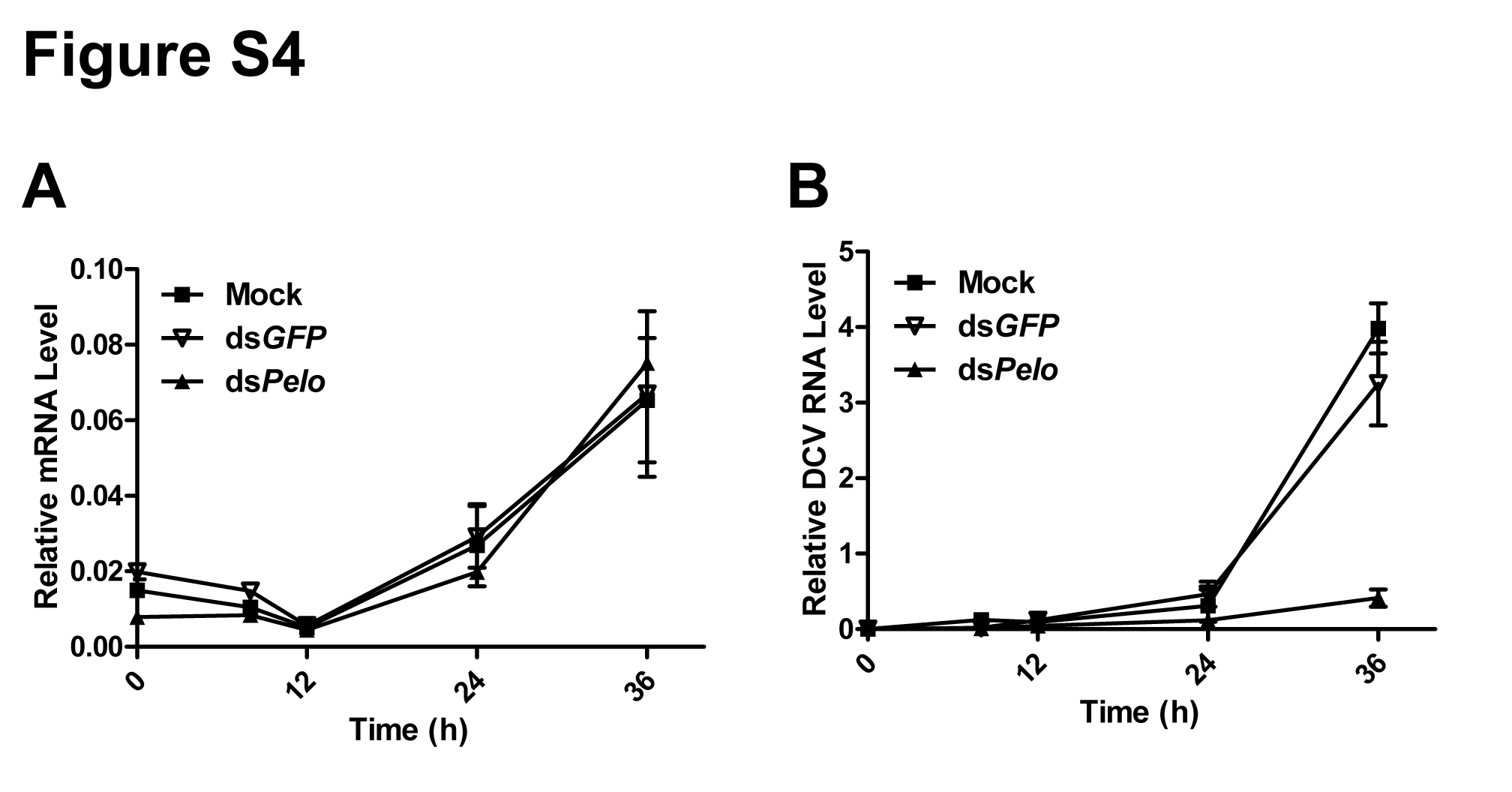

Supplement: Figure S4 — The expressions of JAK-STAT target gene after DCV infection in S2 cells. S2 cells were untreated (Mock) or treated with the indicated dsRNAs for 6 days and then infected with DCV (MOI = 0.1). The expressions of vir-1 (A) and the accumulation of DCV RNA (B) were analyzed by qRT-PCR. Data are the mean ± SD of triplicates. (TIF) [file ppat.1004034.s004.tif]

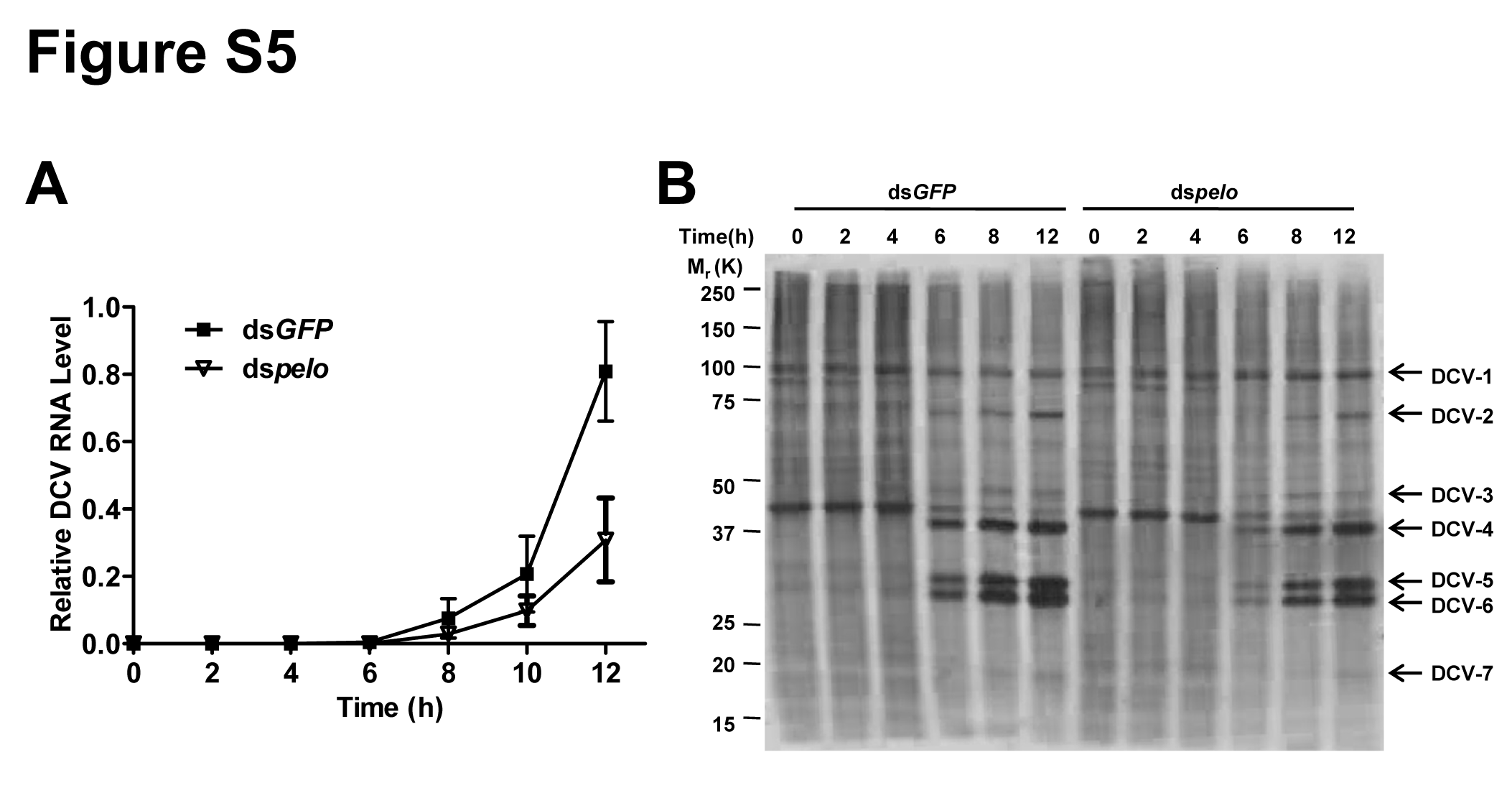

Supplement: Figure S5 — The biosynthesis of viral nucleic acids and proteins during the course of DCV infection. (A) Cells pretreated with the indicated dsRNAs were infected with DCV (MOI = 10) and then collected at different time points. The accumulation of DCV RNA was measured by qRT-PCR. Data are the mean ± SD of triplicates. (B) Cells were labeled with 35S-Met for 30 min at different time points post-infection. Labeled proteins were analyzed by Bis-Tris SDS-PAGE followed by autoradiography. One 35S-Met labeled host cell protein between 37 and 50 KDa was used as loading control for protein analysis. (TIF) [file ppat.1004034.s005.tif]

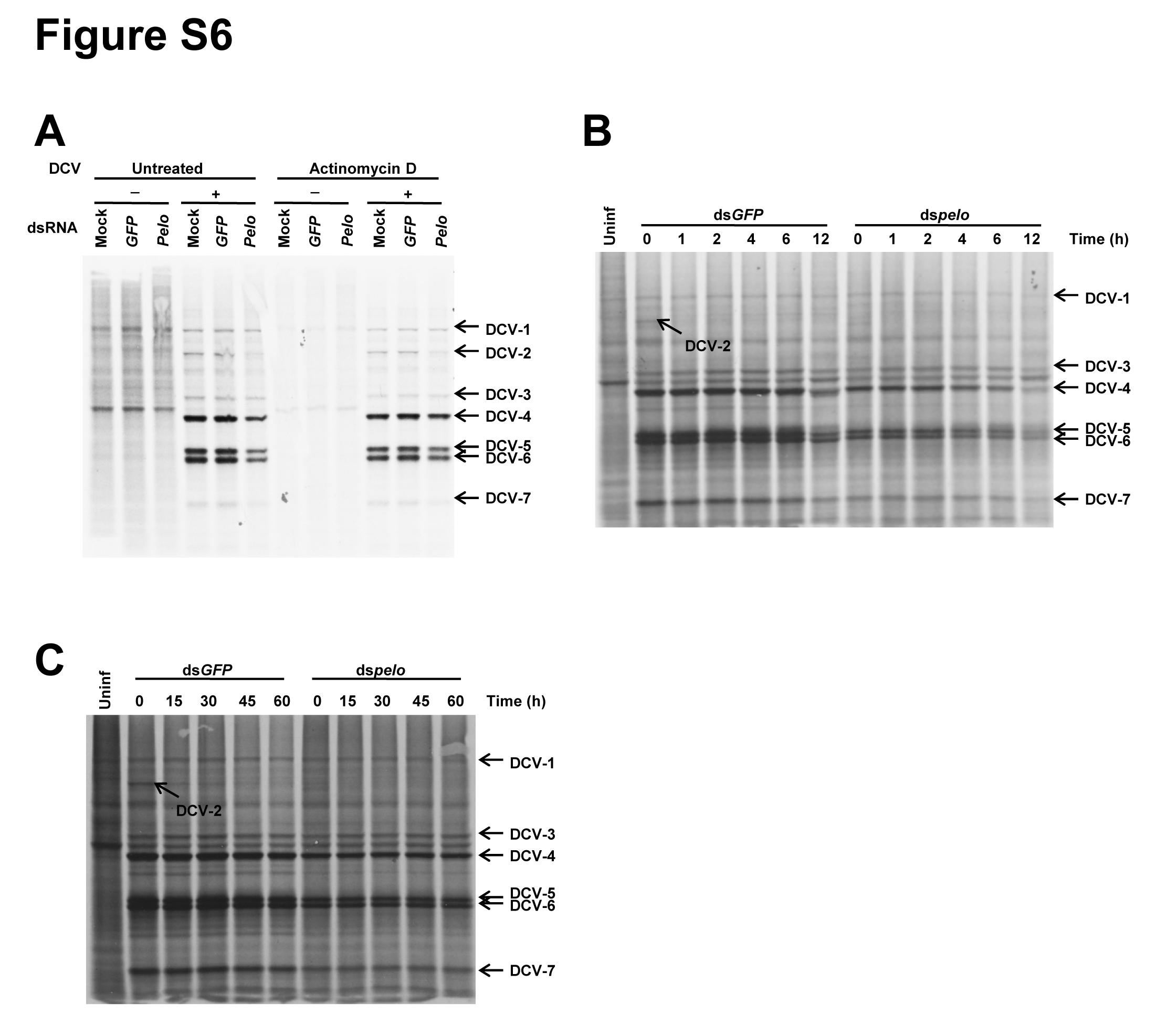

Supplement: Figure S6 — DCV-1 to 7 are viral proteins and DCV-2 has high rate of turnover. (A) Cells pretreated with the indicated dsRNAs were either uninfected or infected with DCV (MOI = 10) for 2 hours and then actinomycin D was added to the medium to inhibit host mRNA transcription. 4 hours later, cells were labeled with 35S-Met for 30 min in the presence of actinomycin D or absence of actinomycin D. Labeled proteins were analyzed by Bis-Tris SDS-PAGE followed by autoradiography. (B and C) Uninfected (Uninf) cells or cells infected with DCV for 6 hours were labeled with 35S-Met for 30 min. Cells were washed three times with PBS and then lysed (time point 0) or chased for the indicated times with excess cold methionine. The stabilities of labeled proteins were detected by Bis-Tris SDS-PAGE, followed by autoradiography. (TIF) [file ppat.1004034.s006.tif]

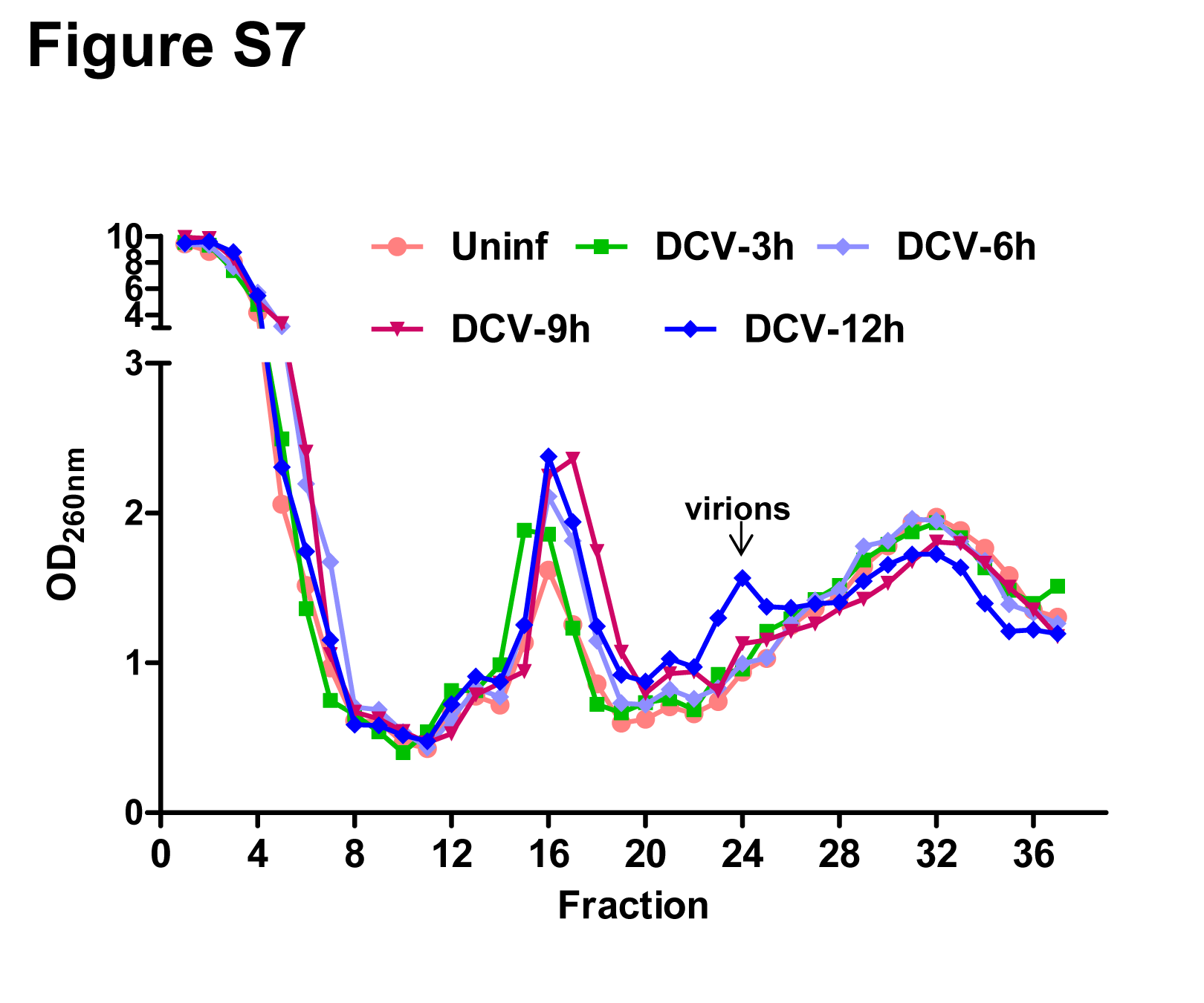

Supplement: Figure S7 — Polysome analysis of DCV infected S2 cells. Cells were infected with DCV (MOI = 10) and then collected at different time points for polysome profile analysis. Lysates were layered on a 10–50% sucrose gradient and centrifuge. 0.3-ml fractions were collected and the polysome profile was monitored by RNA absorbance at 260 nm. Note that there is an extra peak in DCV-infected cells at 12 h post-infection, which is most likely from the packaged viruses. (TIF) [file ppat.1004034.s007.tif]

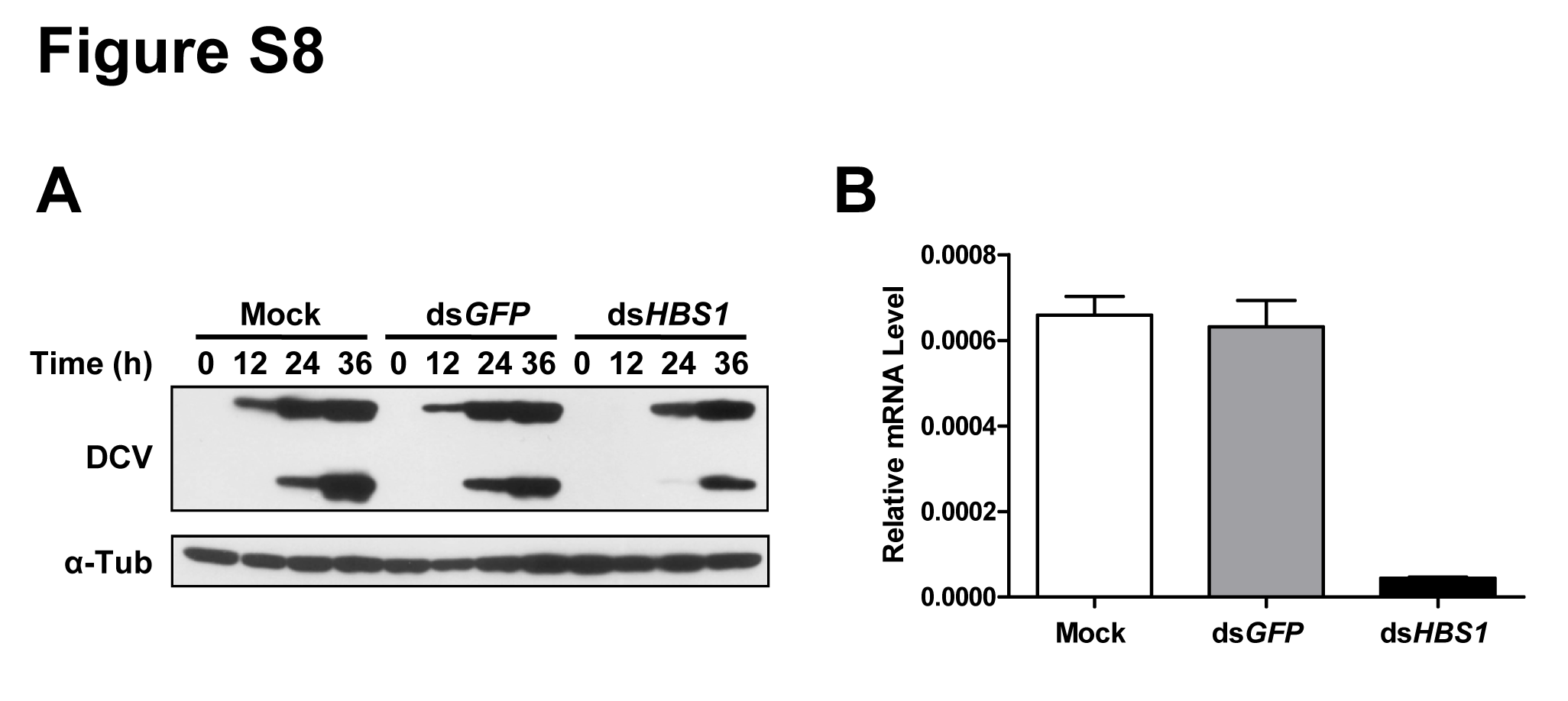

Supplement: Figure S8 — Replication of DCV is slower in HBS1 knockdown S2 cells. (A) Cells pretreated with indicated dsRNAs were challenged with DCV and harvested at different time points post-infection. The accumulations of DCV capsid protein were measured by immunoblotting. (B) Knockdown efficiency of HBS1 was assessed using qRT-PCR. Results are the mean ± SD of triplicates. (TIF) [file ppat.1004034.s008.tif]

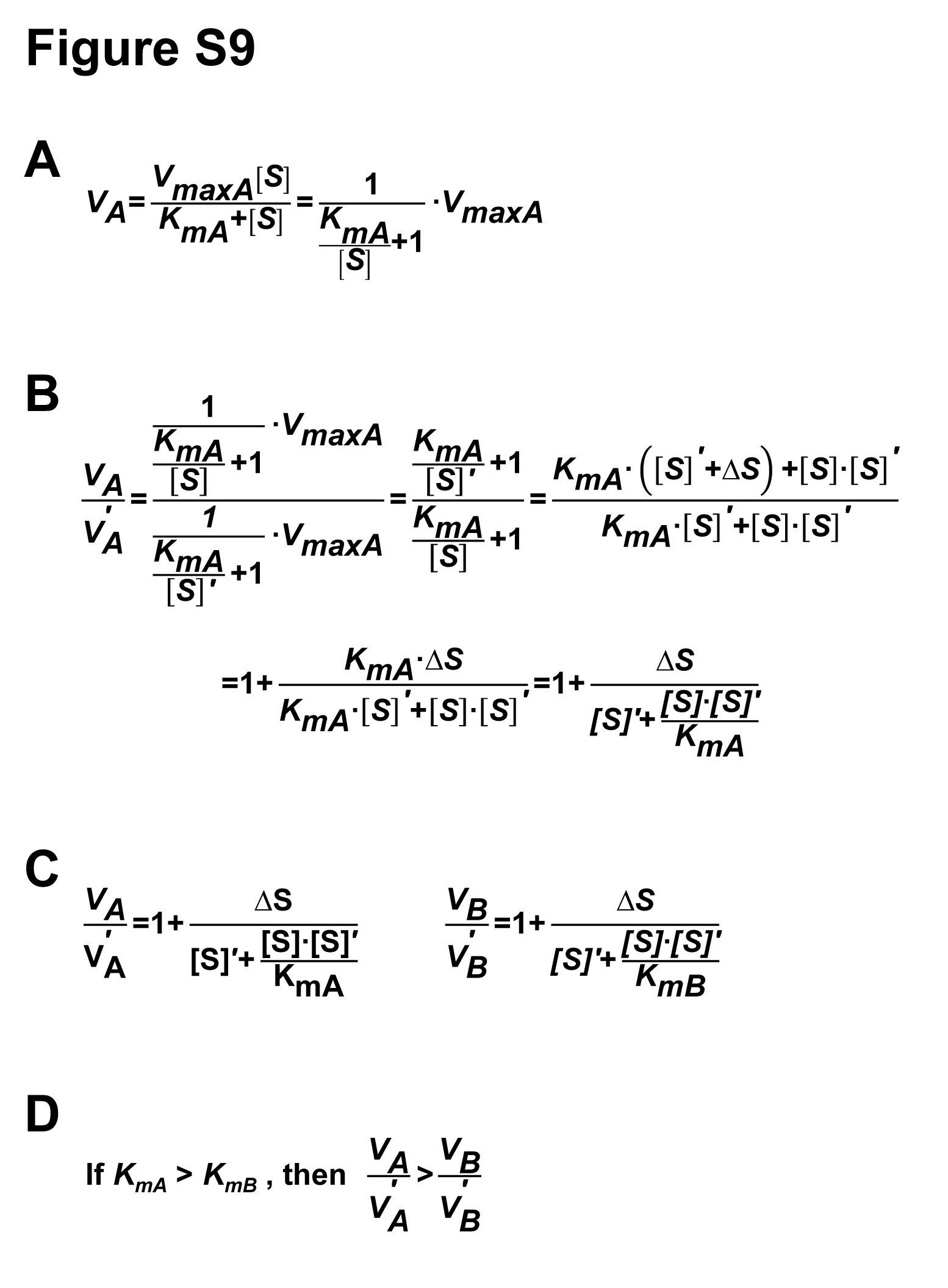

Supplement: Figure S9 — Mathematic calculation of the effect of ribosome concentration on different-speed synthesized proteins. (A) Michaelis-Menten equation of formation of peptide. VA: rate of peptide A formation in wild-type cells. [S]: concentration of free ribosome in wild-type cells. VmaxA: maximum rate of peptide formation. KmA, Michaelis constant of peptide A formation. (B) V′A: rate of peptide A formation in pelo−/− cells. [S]′: concentration of free ribosome in pelo−/− cells. ΔS: [S]-[S]′. (C) VB and VB′: rate of B peptide formation in wild-type and pelo−/− cells, respectively. KmB: Michaelis constant of peptide B formation. (D) The effect of decrease in free ribosome concentration [S] on the more quickly synthesized peptide A is greater than that on the more slowly synthesized peptide B. (TIF) [file ppat.1004034.s009.tif]

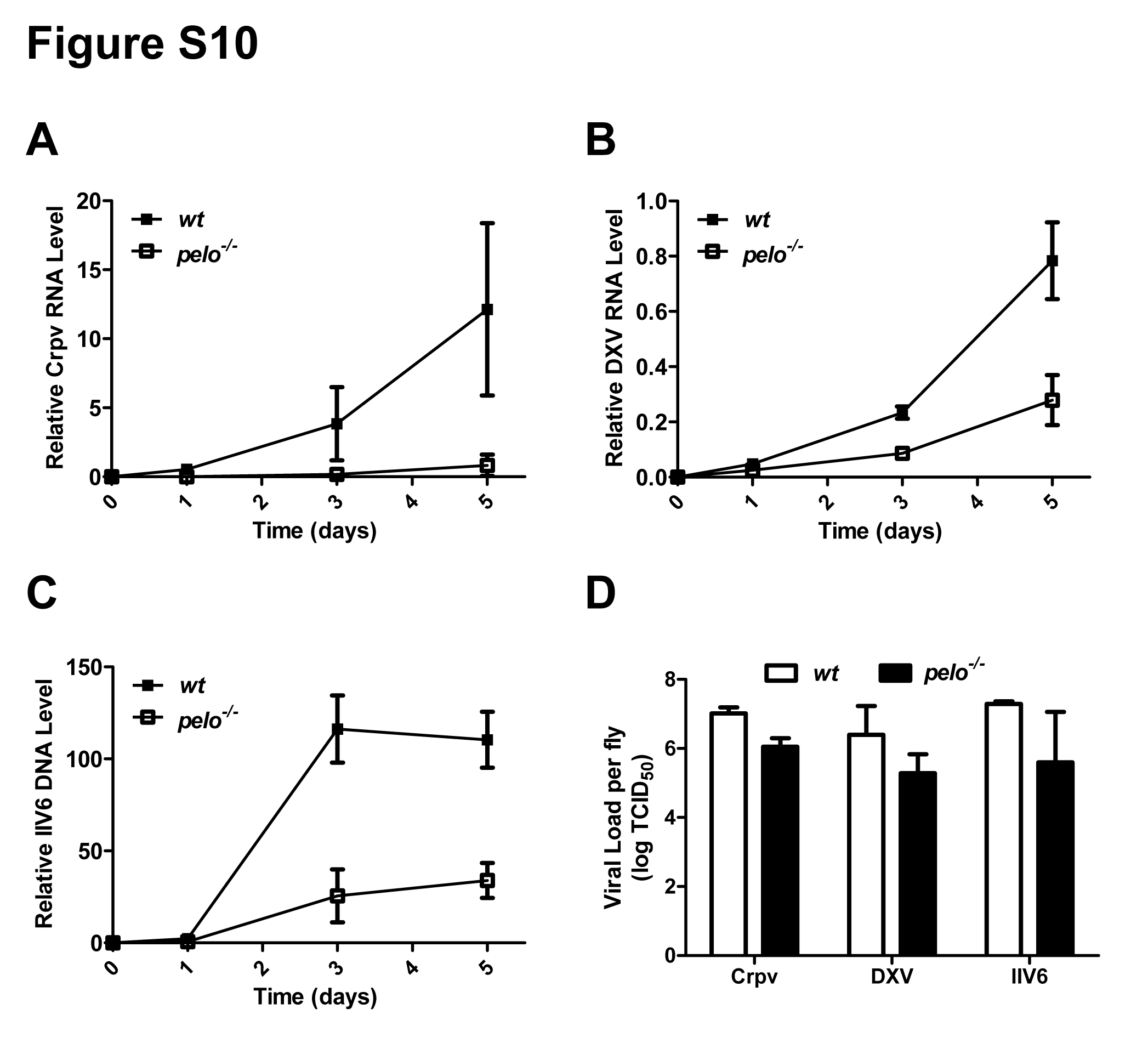

Supplement: Figure S10 — pelo deficiency inhibits the replication of different type viruses. (A–C) wt and pelo−/− flies were infected with virus and collected at the indicated time point. RNA was extracted from Crpv (A) and DXV (B) infected flies, and DNA was isolated from IIV6 (C) infected flies. qRT-PCR or qPCR was used to analyze the amounts of these three different viruses. (D) Flies were infected with indicated virus for 3 days. Three pools of ten flies were collected and homogenized. The viral titer in the homogenate was determined by end-point dilution. Data are the mean ± SD of triplicates. (TIF) [file ppat.1004034.s010.tif]
